# Identification of potential diagnostic and prognostic biomarkers for LUAD based on TCGA and GEO databases

**DOI:** 10.1042/BSR20204370

**Published:** 2021-06-04

**Authors:** Qiangqiang Zheng, Shihui Min, Qinghua Zhou

**Affiliations:** 1 Lung Cancer Center, West China Hospital, Sichuan University, Chengdu, Sichuan 610041, P.R. China; 2 Medical Oncology, West China School of Public Health, Sichuan University, Chengdu, Sichuan 610041, P.R. China; 3 Tianjin Key Laboratory of Lung Cancer Metastasis and Tumor Microenvironment, Tianjin Lung Cancer Institute, Tianjin Medical University General Hospital, Tianjin, China

**Keywords:** clinical features, GEO, hub genes, LUAD, survival, TCGA

## Abstract

Accumulating evidence has demonstrated that gene alterations play a crucial role in LUAD development, progression, and prognosis. The present study aimed to identify the hub genes associated with LUAD. In the present study, we used TCGA database to screen the hub genes. Then, we validated the results by GEO datasets. Finally, we used cBioPortal, UALCAN, qRT-PCR, HPA database, TCGA database, and Kaplan–Meier plotter database to estimate the gene mutation, gene transcription, protein expression, clinical features of hub genes in patients with LUAD. A total of 5930 DEGs were screened out in TCGA database. Enrichment analysis revealed that DEGs were involved in the transcriptional misregulation in cancer, viral carcinogenesis, cAMP signaling pathway, calcium signaling pathway, and ECM–receptor interaction. The combining results of MCODE and CytoHubba showed that ADCY8, ADRB2, CALCA, GCG, GNGT1, and NPSR1 were hub genes. Then, we verified the above results by GSE118370, GSE136043, and GSE140797 datasets. Compared with normal lung tissues, the expression levels of ADCY8 and ADRB2 were lower in LUAD tissues, but the expression levels of CALCA, GCG, GNGT1, and NPSR1 were higher. In the prognosis analyses, the low expression of ADCY8 and ADRB2 and the high expression of CALCA, GCG, GNGT1, and NPSR1 were correlated with poor OS and poor PFS. The significant differences in the relationship of the expression of 6 hub genes and clinical features were observed. In conclusion, 6 hub genes will not only contribute to elucidating the pathogenesis of LUAD and may be potential therapeutic targets for LUAD.

## Introduction

Lung cancer is a common and severe disease which ranks the top among cancers worldwide in terms of mortality for both men and women [1]. In January 2021, the International Agency for Research on Cancer (IARC) published the latest cancer statistics in 2020, according to the statistics published after the investigation of incidence and mortality of 36 cancers in 185 countries, the new cases of lung cancer reached 2.2 million, ranking the second in the number of new cancer cases. About 1.8 million people die from lung cancer, the highest death rate from cancer. In China, the number of new cases and deaths of lung cancer is the highest among all cancers (820 thousand and 710 thousand respectively), accounting for 17.9% and 23.8% of all cancer incidence and mortality [2].

Lung adenocarcinoma (LUAD) is one of the subtypes of non-small cell lung cancer (NSCLC) with high morbidity and mortality [3]. As we all know, the occurrence, development, prognosis, and recurrence of tumors are not only related to the pathological type and clinical stage but also closely related to the expression of tumor genes [4]. With the continuous development of molecular biology and the advocacy of precision medicine, the research and development of targets and targeted drugs in LUAD are becoming more and more mature, and the targeted treatment of LUAD patients is getting more and more attention. A number of genes have been reported to be associated with LUAD, including EGFR, TP53, AKT1, KRAS, and PTEN [5–8]. At present, although the comprehensive treatment of surgery, chemoradiotherapy, targeted therapy, and immunotherapy are applied, the prognosis of LUAD is still poor due to local recurrence or distant metastasis [1]. Therefore, the molecules involved in the carcinogenesis and progression of LUAD need to be further explored, which will contribute to the development of novel treatment strategies of LUAD.

Previous studies have shown that several genetic abnormalities were associated with the initiation and development of LUAD [9,10], but the pathogenesis contributing to biological properties of LUAD remains inconclusive. At present, with the development of high-throughput molecular detection technology, a large volume of disease-associated bioinformatics data has been produced. High-throughput bioinformatics platforms may promote the analysis of differential gene expression, including microarrays, and have a wide range of applications in medical oncology, particularly in searching for disease-associated biomarkers [11], alternative splicing [12], and gene function prediction [13]. Numerous previous studies have generated a large volume of microarray data, and a number of gene expression profiling studies on LUAD have identified differentially expressed genes (DEGs) in various pathways, molecular functions, and biological processes. By bioinformatic analysis of these microarray data, many new genes associated with disease initiation and progression can be found. In the present study, gene expression profile data in LUAD were extracted from TCGA and GEO, and the data used to investigate the potential hub genes in LUAD.

## Materials and methods

### Data collection and processing

High-throughput gene expression data of LUAD tissues and normal lung tissues were extracted from the TCGA Data Portal (https://tcga-data.nci.nih.gov/tcga). These RNA-seq data (HTSeq-count) from Illumina HiSeq RNASeq platform consisted of 502 LUAD samples and 49 adjacent non-cancerous samples, and were achieved from the publicly available Genomic Data Commons (GDC) data portal (https://portal.gdc.cancer.gov/).

### Identification of the aberrantly expressed genes in LUAD

In order to identify the DEGs, R software (version 4.0.5) were applied to compare the expression profiles of LUAD tissues with those of normal tissues. Differential expression analysis of individual genes was carried out by edgeR Bioconductor package [14]. The |log_2_(fold change [FC])| > 2, *P* value < 0.01, and false discovery rate (FDR) < 0.01 were considered as the threshold values for DEG identification. We selected the top 300 up-regulated and 300 down-regulated genes for our study. The heatmap and volcanoplot were performed with ‘gplots' and ’ggplot2’ packages of R software.

### GO and KEGG pathway enrichment analyses of DEGs

The Database for Annotation Visualization and Integrated Discovery (DAVID) [15] is a online tool, which provides a comprehensive set of functional annotation tools for researchers to investigate the biological meaning of genes. To identify the pathways which had the most significant involvement with the genes identified, up-regulated and down-regulated DEGs were submitted into DAVID for Gene ontology (GO) analysis including the biological process (BP), cellular component (CC), and molecular function (MF) enrichment [16] and Kyoto Encyclopedia of Genes and Genomes (KEGG) [17] pathway analysis enrichment, and then visualized in bubble chart by ‘ggplot2’ in R software. *P*<0.05 was considered to indicate a statistically significant difference in the functional enrichment analysis.

### Construction of protein–protein interaction network and hub cluster

Identified DEGs were mapped into the online search tool for the retrieval of interacting genes (STRING, version 11.0) database [17] to evaluate the interactive relationships among the DEGs. Interactions with a combined score > 0.4 were defined as statistically significant. Cytoscape software (version 3.8.2) [18] was used to visualize the integrated regulatory network and mark the high or low expression of gene with different colors and shapes. According to the CytoHubba (version 0.1) plugin in Cytoscape, the top 10 genes according to degree level were selected. In addition, the molecular complex detection (MCODE, version 2.0.0) plugin in cytoscape software was used for the identification of important molecules in PPI networks to screen the modules of hub genes [19].

### Screening the hub genes

The Jvenn, an interactive Venn diagram viewer [20], was used to conduct an intersection analysis to compare the CytoHubba and MCODE plugins in Cytoscape, concurrent genes were defined as hub genes.

### GSEA of the hub genes aberrantly

Gene set enrichment analysis (GSEA, version 4.1.0) was applied to predict associated up-regulated and down-regulated genes and the signifcantly changed pathways based on the expression profle from TCGA database [21]. In each separate analysis, Student’s *t*-test statistical score was conducted in consistent pathways and the mean of the DEGs was calculated. A permutation test with 1000 times was utilized to detect the signifcantly involved hallmark pathways. The adjusted *P*-value using Benjamini and Hochberg (BH) and FDR method by default was used to correct for the occurrence of false positive results. Significant involved genes were defined with an adjusted *P*-value < 0.01 and FDR < 0.25.

### Verification of TCGA results by GEO datasets

To verify the results of TCGA, a search was performed in the Gene Expression Omnibus database (GEO, http://www.ncbi.nlm.nih.gov/geo/). The search strategy was as follows: (‘lung cancer’ OR ‘lung adenocarcinoma’). Gene expression profiles of datasets GSE118370, GSE136043, and GSE140797 were obtained from GEO. The dataset GSE118370 consists of 6 LUAD samples and 6 normal lung samples, GSE136043 consists of 5 LUAD samples and 5 normal lung samples, while GSE140797 consists of 7 LUAD samples and 7 normal lung samples. The data were analyzed on the GPL570 platform Affymetrix Human Genome U133 Plus 2.0 Array and the GPL13497 platform Agilent-026652 Whole Human Genome Microarray 4×44K v2. To identify DEGs between LUAD tissues and normal lung tissues, we employed the Limma package in R software (version 4.0.5) for processing [14]. The adjusted *P*-value was calculated using Benjamini–Hochberg method for controlling the FDR, thus correcting false positives. Cut-off criterion was defined as *P*<0.05 and |log_2_FC| > 2. The platform annotation files downloaded from the database were adopted to convert the probe data in the matrix files into gene symbols. The heatmap and volcanoplot were performed with ‘gplots' and ’ggplot2’ packages of R software. The common expression genes were determined with Jvenn. Then, GO enrichment analysis, KEGG pathway enrichment analysis, and PPI network were performed by the above methods [15–19].

### Potential molecular mechanism of the hub genes in LUAD

It could be assumed that the expression of these genes in LUAD could be caused by genetic alterations, including amplification, deletion, or point mutations. Consequently, cBioPortal [22] was used to summarize the possible genetic alterations for these DEGs in LUAD.

### Transcription level of the hub genes in LUAD

The expression of the hub genes in LUAD tissues and normal lung tissues were analyzed by online software UALCAN (http://ualcan.path.uab.edu/analysis.html). In this study, we analyzed the difference of hub genes expression from different perspectives of primary LUAD, TNM stage, and nodal metastasis status.

### RNA extraction and qRT-PCR of the hub genes

Trizol reagent (Invitrogen, Carlsbad, CA, U.S.A.) was used to extract total RNA from the Beas-2B cells and A549 cells according to the manufacturer’s protocol. Superscript II reverse transcriptase and random primers were used to synthesize cDNA. Quantitative real-time PCR (qRT-PCR) was conducted on the ABI 7900HT Sequence Detection System with SYBR-Green dye (Applied Biosystems, Foster City, CA, U.S.A.). All primers were shown in Table 1. The reaction parameters included a denaturation program (10 min at 95°C), followed by an amplification and quantification program over 45 cycles (15 s at 95°C and 34 s at 60°C). Each sample was tested in triplicates, and each sample underwent a melting curve analysis to check for the specificity of amplification. The expression level was determined as a ratio between the hub genes and the internal control GAPDH in the same mRNA sample, and calculated by the comparative CT method. Expression levels of hub genes were calculated by the 2^−δδCt^ method.

**Table 1:** The primer sequence of qRT-PCR

Gene	Primer	Sequence (5′ to 3′)
ADRB2	Forward	TTGCTGGCACCCAATAGAAGC
	Reverse	CAGACGCTCGAACTTGGCA
ADCY8	Forward	CCTGCGGCACCAAAGTCTT
	Reverse	CGAGTTGCTAGGGGCACAG
CALCA	Forward	ATGGGCTTCCAAAAGTTCTCC
	Reverse	GCCGATGAGTCACACAGGTG
GCG	Forward	CTGAAGGGACCTTTACCAGTGA
	Reverse	CCTGGCGGCAAGATTATCAAG
GNGT1	Forward	ATTACGTTGAAGAACGATCTGGC
	Reverse	GGATGCCCTTTACCAGTGGA
NPSR1	Forward	ATGCCAGCCAACTTCACAGAG
	Reverse	AAGGAGTAGTAGAAGGAACCCC
GAPDH	Forward	GGACCTGACCTGCCGTCTAG
	Reverse	GTAGCCCAGGATGCCCTTGA

### Protein expression level of the hub genes in LUAD

The Human Protein Atlas (https://www.proteinatlas.org/) is a database of immunohistochemistry (IHC)-based protein expression profles in cancer tissues, normal tissues, and cell lines [23]. In the present study, the protein expression IHC images of hub genes in clinical specimens of LUAD patients were obtained from this database.

### Relationship between the hub genes and prognosis of LUAD

The effects of the hub genes were assessed using Kaplan–Meier plotter database (http://kmplot.com) [24]. The Kaplan–Meier plotter database is capable to assess the effect of 54 k genes on survival in 21 cancer types, including breast cancer, ovarian cancer, lung cancer, and gastric cancer. In addition, LUAD patients obtained from TCGA database were divided into the high expression group and the low expression group based on the median expression of hub genes, and the survival curves were drawn with ‘hash’ and ‘survival’ packages of R software.

### Diagnostic role of the hub genes in LUAD

The receiver operating characteristic (ROC) curve was used to assess the diagnostic effectiveness of the hub genes in LUAD. All genes expression data were obtained from TCGA database. ROC curve analysis was performed in R software using procedures from the ‘pROC’ package.

### Relationship between the hub genes and clinicopathological features in LUAD

The clinical data of LUAD were extracted from the TCGA Data Portal (https://tcga-data.nci.nih.gov/tcga). Gene expression data and clinical data were combined by ‘hash’ package of R software, then the clinical data were divided into high expression group and low expression group according to the median of expression level of hub genes. The relationship between the expression levels of hub genes and clinical characteristics was analyzed by statistical analysis of the clinical characteristics of high expression group and low expression group, and visualized in forestplot by ‘forestplot’ package of R software. Statistical analyses were performed using Pearson’s chi-squared test in SPSS (version 22.0). The odds ratio (OR) and corresponding 95% confidence interval (CI) of each factor were calculated by STATA (version 14.0) software. Data were considered statistically significant when *P*<0.05.

## Results

### Aberrantly expressed genes based on TCGA data in LUAD

**Figure 1:**
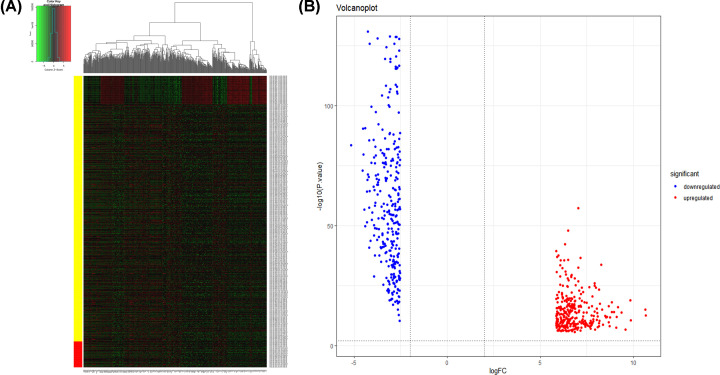
Heatmap and Volcanoplot of the distribution of DEGs between LUAD tissues and normal lung tissues (**A**) Heatmap of 600 DEGs between LUAD tissues and normal lung tissues. The up-regulated and down-regulated genes were shown in red and green, respectively. (**B**) Volcanoplot of 600 DEGs between LUAD tissues and normal lung tissues. The up-regulated and down-regulated genes were shown in red and blue, respectively.

The expression level of each gene transformed with log2 was calculated by EdgeR. Following the calculating criteria, we achieved 5930 aberrantly expressed genes in LUAD, including 5208 highly and 722 lowly expressed genes. Then we listed the top 300 up-regulated and 300 down-regulated genes according to the value of |log_2_FC| (Table 2), which demonstrated that these genes might play vital roles in the occurrence of LUAD. The ‘gplots' and ’ggplot2’ packages of R software were used to draw heatmap and volcanoplot of the 600 genes (Figure 1).

**Table 2:** Compared with adjacent non-tumorous tissues, the top 300 up-regulated and 300 down-regulated genes in LUAD based on TCGA database

DEGs	Genes
Up-regulated	TFF2, REG4, LINC00676, FGB, DEFA5, MAGEA3, ALB, MAGEA6, RNU5B-1, MAGEC1, CALCA, TFF1, MAGEA10, MAGEA4, LINC01419, FGF19, AFP, CGA, MAGEA12, PRB4, SPINK4, PITX2, TFAP2D, CASP14, PSG1, GC, BARX1, GCG, INSL4, ACTL8, PDX1, HOXC12, C11orf86, TRIM48, FGL1, CSAG1, PRAME, LINC02418, LIN28B, KRT20, CPN1, PAEP, SERPINA4, TM4SF5, DSCAM-AS1, RNU5A-1, MAGEA1, DCAF4L2, RN7SKP255, RNY1, MAGEC2, PSG4, PSG5, COX7B2, SPP2, HIST1H1B, PAGE2, REG1A, F2, AKR1B10, PSG3, CGB5, SST, SPANXB1, CALML5, DLK1, HOXB9, SSX1, PADI3, UGT1A10, AC079466.1, NKX1-2, UGT2B11, SNORA71D, RNU4-1, DLL3, HOXC13, MAGEA11, MUC17, KRTAP4-1, APOBEC1, ANXA10, FOXD3-AS1, SPAG11B, OTX2, TPSP2, VIL1, AC026785.3, LHX1, HIST1H3C, BARX1-DT, FAM83A, TRPM8, GAGE2A, CYP24A1, AL160271.1, MAGEA9B, RNY3, PSG11, RF00100, PAX7, MAGEB2, SNORA74A, SPINK1, HIST1H4L, AL136537.2, IGF2BP1AC011298.1, HOXC11, HIST1H1D, AC007128.2, VGLL2, HOTAIR, NEUROD1, SPAG11A, MAGEB1, SLCO1B3, KRT81, PCSK1, POU3F2, RNU4-2, UGT1A8, LINC01667, CYP11B1, HIST1H3B, HIST1H3F, KLK6, DSCR8, BPIFB4, RNA5-8SP2, SNORA23, KRT6A, AC079062.1, CPS1, AC090502.1, CST4, LINC00942, AL513304.1, TMPRSS11E, AC133785.1, SP3P, HOXD13, EPS8L3, SP8, SNORA73B, AF274573.1, HIST1H2AJ, ASNSP1, SCARNA5, AC005256.1, TINAG, ANKS4B, AC061975.6, DPYSL5, PASD1, PHOX2B, FOXI3, SPRR1B, MMP13, SLCO1B1, BCAR4, CT83, KCNC2, MIR548XHG, FTHL17, RN7SKP203, FAM83A-AS1, ONECUT1, LIPK, GNGT1, INHA, LIN28A, AC114786.2, EEF1A2, HIST1H2BI, LINC01234, AC133681.1, IL37, SLC18A3, AL355075.4, FAR2P1, CLDN10-AS1, TCN1, NR0B1, MAGEA4-AS1, HOXC13-AS, LHX1-DT, LINC01833, AL354685.1, HOXA13, FUT9, TUBA3C, AL139023.1, PRSS1, MYEOV, DMBX1, HIST1H4F, SNORA49, AFAP1-AS1, AC098592.1, ADGRG7, GFY, LINC01559, PADI1, HOXB13, ERVV-2, LINC00973, POU6F2-AS2, AC026336.3, CALML3, AC023824.1, FEZF1, AS1, KRT75, SLC2A2, SCARNA10, KLK12, DPPA2, TM4SF20, LINC02163, LINC01980, HIST1H2BM, HMGA2, AL121949.2, VGF, AKR1B15, LINC01194, COL11A1, CTAG2, MAGEB16, SOX14, TRIM15, CST1, INS, IL36RN, VCX3A, ETNPPL, S100A7, PAH, OLFM4, LINC01468, GAGE1, AC016044.1, BRDT, Z98257.1, HOXC10, LINC01518, IL1RAPL2, HIST1H2BB, HHIPL2, CRABP1, RN7SKP9, KISS1R, ZNF280A, AC079160.1, BAGE2, SNORA54, MIR3609, RMRP, TEX19, DUSP13, PRB1, HIST1H4C, PIWIL3, FAM230C, AC020907.1, RN7SKP227, UGT3A1, GPX2, C6orf15, DKK4, HOXD11, UCA1, G6PC, LHX5, AC106785.2, LINC01511, EIF4E1B, LINC01214, ATP10B, NPSR1, AF127577.3, PCDH8, PPY2P, KCNU1, GABRA3, AP003900.1, PPP1R14D, PSG6, PRSS2, KIF1A, DSCR4, FETUB, MUC6, FABP7, PNPLA5, SNORA80A, SALL3, SP9, AC068228.1, HIST1H3J
Down-regulated	SLC6A4, CD300LG, AGER, AL606469.1, HTR3C, FABP4, SFTPC, LINC02016, CSF3, CHRM1, MYOC, FAM107A, ANKRD1, HBA1, AC095050.1, SERTM1, OR6K3, ANGPT4, ITLN2, FCN3, GPM6A, LGI3, LINC01996, TMEM100, CA4, AC084030.1, ALAS2, AP002856.2, RPL13AP17, UPK3B, AC128709.3, AC093787.1, AC104984.1, HBB, AC008763.3, MCEMP1, WNT3A, GPD1, HBA2, SH3GL3, AC135012.3, AC009093.3, NCAPGP2, RTKN2, LINC01082, CD5L, OVCH1, C10orf67, FENDRR, FREM3, PI16, AC104984.4, CLEC4M, AL354714.1, TNR, KCNA4, GPIHBP1, CLDN18, AC010776.3, MTATP8P1, AL590226.1, GRIA1, OTC, SGCG, ANGPTL7, CLEC3B, SOSTDC1, SSTR4, ADAMTS8, CHRNA2, CHIAP2, RS1, ADAMTS7P3, ARMH2, ARC, SCUBE1, VEGFD, CLIC5, LINC00656, AC104237.2, KRT79, MIR3945HG, EDNRB, AC104237.3, LANCL1, AS1, AC026369.3, PTPRQ, AL445307.1, NCKAP5, FHL1, INMT, BTNL9, CHRM2, PRX, CACNA1S, ADRA1A, LINC00968, TNNC1, HAS1, CYP1A2, DNASE1L3, LINC00163, STXBP6, CNTN6, GP9, ADH1B, INMT-MINDY4, MYZAP, PACRG-AS3, LINC01070, SCGB1A1, AC013457.1, CAV1, CAVIN2, Z82246.1, CTXND1, DES, AL772337.2, SLC4A1, AC104257.1, LINC01081, FMO2, LINC02570, GDF10, ADCY8, AC093110.1, LINC02154, GYPE, VIPR1, SEMA3G, AC044810.2, AC091305.1, TEK, ST8SIA6, SPOCK2, RAMP3, MGAT3, DEFA3, DNASE2B, CAV3, TCF21, AGBL1, ADRB1, NECAB1, ECEL1P2, ALKAL2, MYH2, FOLR3, HELT, LINC00551, ODAM, ACADL, AL591686.1, RETN, LINC01616, RSPO1, DPP6, AL136369.1, FGF10, MARCO, PCDH15, AL161740.1, LYVE1, AC027288.3, AL359378.1, FOSB, CCDC141, PTPN5, AGRP, CMTM5, OVCH2, AC004947.1, AC113404.1, RXFP1, FGF10-AS1, LINC01863, FXYD1, AC092691.1, PARAL1, FPR2, C2orf71, AP000438.1, MT1JP, ADRB2, SEC14L3, AC053503.6, SFRP5, AC010976.2, FGFBP2, GPA33, STX11, IL1RL1, AC119424.1, AQP4, SOX7, ADGRE3, AC027288.2, SCN4B, SLC14A1, SLC19A3, ADGRG4, FAM189A2, AC096531.2, ABCA8, PPP1R17, AC005324.1, WFIKKN2, AP001189.1, COL6A6, RAMP2, RGCC, IGSF10, LRRC36, ROBO4, CD36, KANK3, CD101, AL355974.2, SIRPB1, AL109741.3, FO681492.1, S1PR1, HHIP, ACTN2, LIMS2, HID1-AS1, AC116312.1, FHL5, RBP2, CXCR1, LINC00844, TAL1, ANOS1, AC092834.1, AL354714.3, CASQ2, FOXF1, HIGD1B, HPSE2, LINC01447, TNXB, HSPA12B, TENT5B, PLAC9, MASP1, SERPINA2, SFTPA1, EDN3, C2orf91, CCDC85A, MT1M, AWAT2, SOX17, EMCN, RXFP2, PLA2G4F, LRRN3, AC093890.1, NMUR1, MAMDC2, TUBB1, MFAP4, AP003385.4, AC026992.2, LAMP3, F11, AC104211.2, CLDN5, SCARA5, CALCRL, AC105914.2, SPAAR, NAV2-AS2, PLAC9P1, AOC3, ASPA, EMP2, AC010776.2, AL138737.1, CATSPERD, EPAS1, DCC, GSG1L, MOGAT1, AC097537.1, LINC01645, PLPPR5, AC018647.1, AC134312.1, CDO1, C14orf132, TGFBR3, NPR1, CCL24, GCOM1, MGAT3-AS1, JPH4, PRKG2, CRTAC1, ARHGEF26

### GO function and KEGG pathway analyses for the DEGs

**Figure 2:**
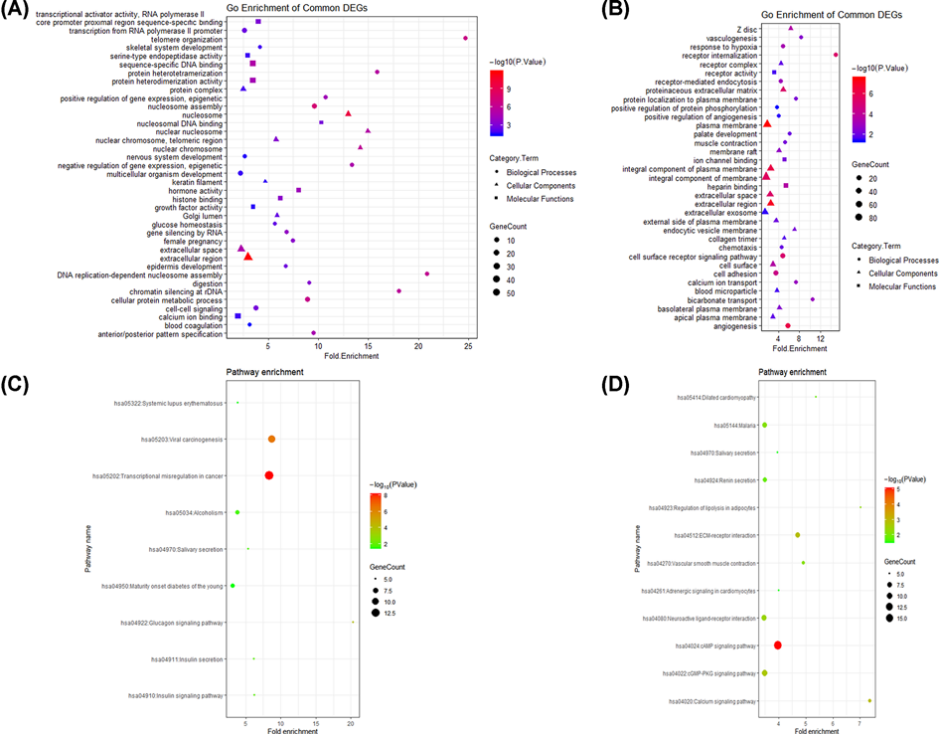
GO and KEGG functional enrichment analyses of 300 up-regulated and 300 down-regulated DEGs in LUAD (**A**) GO analysis of 300 up-regulated DEGs, (**B**) GO analysis of 300 down-regulated DEGs, (**C**) KEGG functional enrichment analysis of 300 up-regulated DEGs, (**D**) KEGG functional enrichment analysis of 300 down-regulated DEGs. Circle, triangle, and square represented BP or KEGG functional enrichment, CC, and MF, respectively. The size of the dot represented gene count, and the color of the dot represented the *P* value.

To identify the pathways which had the most significant involvement with the genes identified, enriched GO categories and KEGG pathways were identified by uploading selected DEGs to DAVID. The results of the GO analysis indicated that in biological process terms, the up-regulated DEGs were mainly enriched in nucleosome assembly, telomere organization, and cellular protein metabolic process (Figure 2A, Table 3), down-regulated DEGs were significantly enriched in angiogenesis, receptor internalization, and cell surface receptor signaling pathway (Figure 2B, Table 3). In cell component terms, up-regulated DEGs were mainly enriched in extracellular region, nucleosome, and nuclear chromosome (Figure 2A, Table 3), whereas down-regulated DEGs were mainly enriched in plasma membrane, extracellular region, and integral component of plasma membrane (Figure 2B, Table 3). In molecular function terms, up-regulated DEGs were mainly enriched in sequence-specific DNA binding, protein heterodimerization activity, and hormone activity (Figure 2A, Table 3), whereas down-regulated DEGs were mainly enriched in heparin binding, ion channel binding, and receptor activity (Figure 2B, Table 3).

**Table 3:** GO analysis and KEGG pathway analysis of the top 300 up-regulated and 300 down-regulated genes associated with LUAD

Category	GO ID	Term	Count	*P* value
**A. Up-regulated DEGs**				
BP	GO:0006366	Transcription from RNA polymerase II promoter	14	0.003
BP	GO:0007275	Multicellular organism development	12	4.58E-08
BP	GO:0006334	Nucleosome assembly	12	0.021
BP	GO:0044267	Cellular protein metabolic process	11	4.32E-07
BP	GO:0007267	Cell–cell signaling	10	0.001
BP	GO:0007399	Nervous system development	8	1.98E-05
BP	GO:0031047	Gene silencing by RNA	8	1.62E-04
BP	GO:0009952	Anterior/posterior pattern specification	8	0.031
BP	GO:0007565	Female pregnancy	7	2.90E-07
BP	GO:0045815	Positive regulation of gene expression, epigenetic	7	8.51E-07
BP	GO:0045814	Negative regulation of gene expression, epigenetic	7	2.09E-06
BP	GO:0051290	Protein heterotetramerization	7	4.52E-06
BP	GO:0000183	Chromatin silencing at rDNA	7	1.28E-05
BP	GO:0006335	DNA replication-dependent nucleosome assembly	7	4.46E-05
BP	GO:0032200	Telomere organization	7	3.34E-04
BP	GO:0007596	Blood coagulation	6	5.01E-04
BP	GO:0001501	Skeletal system development	6	0.002
BP	GO:0042593	Glucose homeostasis	6	0.004
BP	GO:0008544	Epidermis development	6	0.014
BP	GO:0007586	Digestion	6	0.044
CC	GO:0005576	Extracellular region	51	2.03E-12
CC	GO:0005615	Extracellular space	33	1.32E-05
CC	GO:0000786	Nucleosome	13	3.16E-10
CC	GO:0043234	Protein complex	11	0.013
CC	GO:0000784	Nuclear chromosome, telomeric region	8	1.37E-06
CC	GO:0000228	Nuclear chromosome	8	4.73E-04
CC	GO:0000788	Nuclear nucleosome	7	6.59E-06
CC	GO:0005796	Golgi lumen	6	0.004
CC	GO:0045095	Keratin filament	5	0.022
MF	GO:0043565	Sequence-specific DNA binding	19	8.84E-06
MF	GO:0046982	Protein heterodimerization activity	17	3.21E-05
MF	GO:0005509	Calcium ion binding	15	0.019
MF	GO:0001077	Transcriptional activator activity, RNA polymerase II core promoter proximal region sequence-specific binding	10	8.79E-04
MF	GO:0004252	Serine-type endopeptidase activity	8	5.88E-05
MF	GO:0042393	Histone binding	8	3.01E-04
MF	GO:0005179	Hormone activity	8	0.018
MF	GO:0008083	Growth factor activity	6	0.028
MF	GO:0031492	Nucleosomal DNA binding	5	0.001
KEGG	hsa05202	Transcriptional misregulation in cancer	14	5.84E-09
KEGG	hsa05203	Viral carcinogenesis	11	3.48E-07
KEGG	hsa05034	Alcoholism	6	0.019
KEGG	hsa04950	Maturity onset diabetes of the young	6	0.041
KEGG	hsa04922	Glucagon signaling pathway	5	8.74E-05
KEGG	hsa04910	Insulin signaling pathway	5	0.008
KEGG	hsa04911	Insulin secretion	5	0.008
KEGG	hsa04970	Salivary secretion	5	0.013
KEGG	hsa05322	Systemic lupus erythematosus	5	0.039
**B. Down-regulated DEGs**				
BP	GO:0007155	Cell adhesion	17	3.85E-05
BP	GO:0007166	Cell surface receptor signaling pathway	14	9.13E-07
BP	GO:0001525	Angiogenesis	14	8.83E-06
BP	GO:0006898	Receptor-mediated endocytosis	9	5.60E-04
BP	GO:0001666	Response to hypoxia	9	9.35E-04
BP	GO:0031623	Receptor internalization	7	6.32E-06
BP	GO:0006935	Chemotaxis	6	0.001
BP	GO:0006936	Muscle contraction	6	0.006
BP	GO:0006816	Calcium ion transport	6	0.010
BP	GO:0001934	Positive regulation of protein phosphorylation	5	0.001
BP	GO:0045766	Positive regulation of angiogenesis	5	0.003
BP	GO:0060021	Palate development	5	0.005
BP	GO:0072659	Protein localization to plasma membrane	5	0.009
BP	GO:0001570	Vasculogenesis	5	0.036
BP	GO:0015701	Bicarbonate transport	5	0.049
CC	GO:0016021	Integral component of membrane	86	3.44E-06
CC	GO:0005886	Plasma membrane	79	3.44E-08
CC	GO:0005576	Extracellular region	42	1.29E-07
CC	GO:0070062	Extracellular exosome	41	0.034
CC	GO:0005887	Integral component of plasma membrane	37	9.02E-07
CC	GO:0005615	Extracellular space	34	6.22E-06
CC	GO:0009986	Cell surface	17	2.48E-04
CC	GO:0005578	Proteinaceous extracellular matrix	14	6.55E-06
CC	GO:0016324	Apical plasma membrane	9	0.002
CC	GO:0045121	Membrane raft	9	0.013
CC	GO:0009897	External side of plasma membrane	8	2.79E-04
CC	GO:0016323	Basolateral plasma membrane	8	0.003
CC	GO:0030018	Z disc	8	0.008
CC	GO:0072562	Blood microparticle	6	0.012
CC	GO:0043235	Receptor complex	6	0.024
CC	GO:0005581	Collagen trimer	5	0.005
CC	GO:0030666	Endocytic vesicle membrane	5	0.017
MF	GO:0008201	Heparin binding	9	2.46E-04
MF	GO:0004872	Receptor activity	7	0.025
MF	GO:0044325	Ion channel binding	6	0.006
KEGG	hsa04024	cAMP signaling pathway	16	8.29E-06
KEGG	hsa04022	cGMP-PKG signaling pathway	10	0.002
KEGG	hsa04080	Neuroactive ligand-receptor interaction	9	0.004
KEGG	hsa04512	ECM–receptor interaction	8	0.001
KEGG	hsa05144	Malaria	8	0.008
KEGG	hsa04924	Renin secretion	7	0.014
KEGG	hsa04020	Calcium signaling pathway	6	0.001
KEGG	hsa04270	Vascular smooth muscle contraction	6	0.007
KEGG	hsa04923	Regulation of lipolysis in adipocytes	5	0.005
KEGG	hsa05414	Dilated cardiomyopathy	5	0.013
KEGG	hsa04261	Adrenergic signaling in cardiomyocytes	5	0.035
KEGG	hsa04970	Salivary secretion	5	0.036

KEGG pathway analysis demonstrated that up-regulated DEGs were enriched in transcriptional misregulation in cancer, viral carcinogenesis, and glucagon signaling pathway (Figure 2C, Table 3), whereas down-regulated DEGs were significantly enriched in cAMP signaling pathway, calcium signaling pathway, and ECM–receptor interaction (Figure 2D, Table 3).

### PPI network construction and module analysis of DEGs

**Figure 3:**
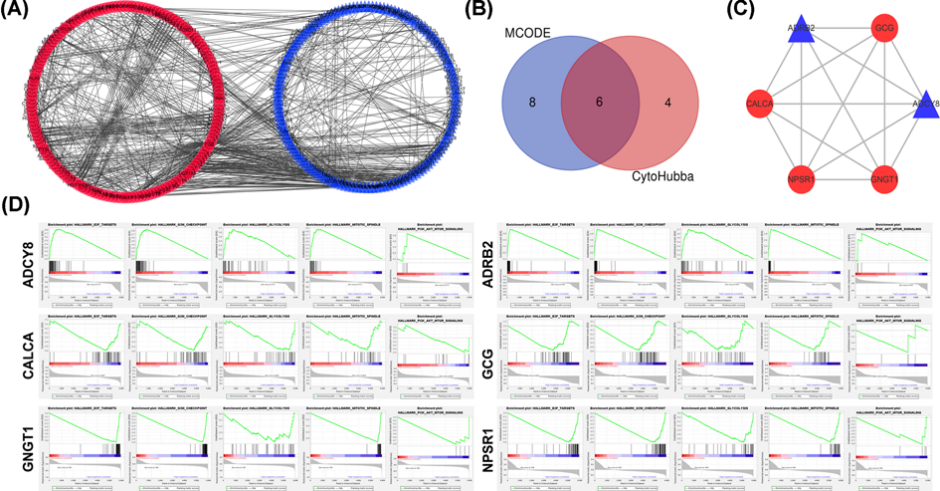
The 600 DEGs in LUAD were analyzed to identify the hub genes (**A**) PPI network of DEGs. The red circle represented up-regulated genes, and the blue triangle represented down-regulated genes. (**B**) The Venn diagram was used to screen common DEGs calculated using two plugins of Cytoscape (CytoHubba and MCODE). (**C**) MCODE identification of the most significantly enriched module. The red circle represented up-regulated genes, and the blue triangle represented down-regulated genes. (**D**) GSEA of the 6 hub genes.

Interactions between the identified DEGs were revealed by constructing a PPI network. In total, there were 414 nodes and 596 edges in the network (Figure 3A). Subsequently, CytoHubba plugin was used to identify the 10 hub nodes with the highest degrees (Table 4), including albumin gene (ALB, score: 34), proglucagon gene (GCG, score: 30), transducin γ-subunit gene (GNGT1, score: 28), insulin (INS, score: 26), adenylate cyclase 8 (ADCY8, score: 23), coagulation factor II (F2, score: 23), neuropeptide S receptor 1 (NPSR1, score: 22), adrenoceptor beta 2 (ADRB2, score: 18), calcitonin-related polypeptide alpha (CALCA, score: 16), and somatostatin receptor (SST, score: 16). Then, a significant module was subsequently constructed with 14 nodes, which gained the highest MCODE score (Table 4). After combining the results of MCODE and CytoHubba plugins, 6 hub genes were determined, including ADCY8, ADRB2, CALCA, GCG, GNGT1, and NPSR1 (Figure 3B). The 6 hub genes were loaded into the STRING database, to obtain the PPI data among them, and PPIs with highest interaction score (confidence > 0.4) were selected. In total, there were 6 nodes and 14 edges in the network (Figure 3C).

**Table 4:** The Venn analysis result of Cytoscape plugins CytoHabba and MCODE

Names	Total	Elements
CytoHubba and MCODE	6	ADCY8, ADRB2, CALCA, GCG, GNGT1, NPSR1
CytoHubba	14	ADCY8, ADRB1, ADRB2, CALCA, CALCRL, GCG, CGA, GNGT1, NPSR1, RAMP2, RAMP3, RXFP1, RXFP2, VIPR1
MCODE	10	ADCY8, ADRB2, ALB, CALCA, F2, GCG, GNGT1, INS, NPSR1, SST

GSEA enrichment analysis, including ADCY8, ADRB2, CALCA, GCG, GNGT1, and NPSR1 indicated that the low expression of ADCY8 and ADRB2 and the high expression of CALCA, GCG, GNGT1, and NPSR1 were positively correlated with E2F targets, G2M checkpoint, glycolysis, mitotic spindle, and PI3K-Akt-mTOR signaling. The details were illustrated in Figure 3D.

### The verification results of GEO database

**Figure 4:**
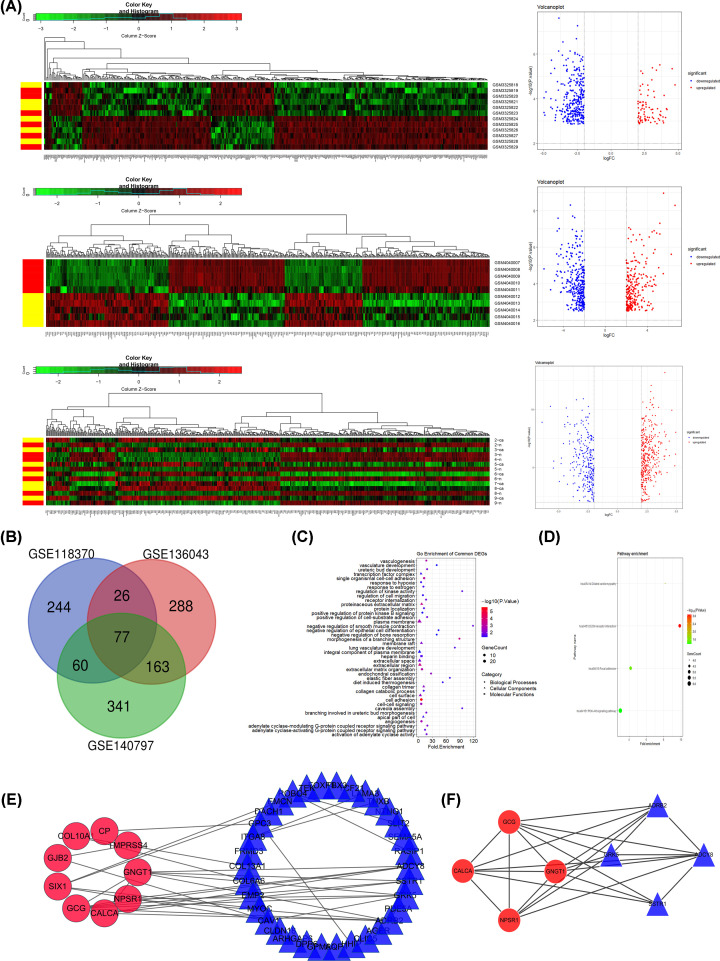
The verification results of GEO database (**A**) Heatmaps and Volcanoplots of the distribution of DEGs in GSE118370, GSE136043, and GSE140797 datasets. The up-regulated and down-regulated genes were shown in red and green or blue, respectively. (**B**) Venn diagram showing number of common genes from GSE118370, GSE136043, and GSE140797 datasets. (**C**) GO analysis of 77 DEGs. Circle, triangle, and square represented BP, CC, and MF, respectively. the size of the dot represented gene count, and the color of the dot represented the *P* value. (**D**) KEGG functional enrichment analysis of 77 DEGs. The size of the dot represented gene count, and the color of the dot represented the *P* value. (**E**) PPI network of the identified 77 DEGs. The red circle represented up-regulated genes, and the blue triangle represented down-regulated genes. (**F**) MCODE identification of the most significantly enriched module. The red circle represented up-regulated genes, and the blue triangle represented down-regulated genes.

This section included three gene sets (GSE118370, GSE136043, and GSE140797), of which GSE118370 included 407 DEGs, GSE136043 included 554 DEGs, and GSE140797 included 641 DEGs. The heatmaps and volcanoplots of above three gene sets were shown in Figure 4A. In all included datasets, compared with normal samples, there are 77 common DEGs (Figure 4B, Table 5). By using DAVID, we found that these DEGs were mainly enriched in cell adhesion, morphogenesis of a branching structure, single organismal cell–cell adhesion, extracellular region, proteinaceous extracellular matrix, plasma membrane, heparin binding, etc. (Figure 4C, Table 6). At the same time, the analysis of the KEGG pathway showed that 77 DEGs were mainly enriched in 4 pathways, namely, ECM–receptor interaction, dilated cardiomyopathy, focal adhesion, and PI3K-Akt signaling pathway (Figure 4D, Table 6). By using the STRING database and Cytoscape 3.8.2 software, a PPI network was constructed for the 77 DEGs. The PPI network had a total of 77 nodes and 60 edges, and an interaction score > 0.4 was considered a high-confidence interaction relationship (Figure 4E). Using Cytoscape plugin MCODE, we identified the top 8 genes with the most connectedness (Figure 4F), including ADCY8, ADRB2, CALCA, GCG, GNGT1, GRK5, NPSR1, and SSTR1. The 8 connected genes contained the 6 hub genes that we had determined in TCGA database.

**Table 5:** The Venn analysis result of GSE118370, GSE136043, and GSE140797 datasets in GEO database

Names	Total	Elements
GSE118370, GSE136043, GSE140797	77	ABCC3, ACADL, ADCY8, ADH1B, ADRB2, AGER, AGR2, ANKRD29, AOC3, AQP4, ARHGAP6, ASPA, BTNL9, CALCA, CAV1, CCDC85A, CD24, CD36, CDO1, CLDN18, CLIC5, COL10A1, COL13A1, COL6A6, CP, DACH1, DPP6, EMCN, EMP2, FLJ34503, FMO2, FOXF1, FRMD3, GCG, GJB2, GNGT1, GPC3, GPM6A, GRK5, HHIP, IGSF10, ITGA8, KANK3, KIAA1462, LAMA3, LDB2, LOC400568, MFAP4, MYOC, MYZAP, NPSR1, NTNG1, PDE5A, PKHD1L1, RASIP1, RBP2, ROBO4, RTKN2, SCN7A, SEMA5A, SGCG, SGPP2, SIX1, SLIT2, SPINK1, SPOCK2, SSTR1, SVEP1, TBX3, TCF21, TEK, TMEM100, TMPRSS4, TNXB, TTN, VGLL3, WFDC2
GSE118370	407	ABCA6, ABCA9, ABCC3, ABI3BP, ACADL, ACE, ACSS3, ACTN2, ACVRL1, ADAMTS17, ADAMTS7, ADARB1, ADCY8, ADGRL2, ADGRL3, ADH1B, ADRA1A, ADRB2, AFAP1-AS1, AFAP1L1, AGAP11, AGER, AGR2, AKAP12, AKAP2, ALX1, AMER2, ANGPT1, ANKRD29, ANXA8L1, AOC3, APOL3, AQP3, AQP4, ARHGAP29, ARHGAP6, ARHGEF15, ARHGEF26, ARHGEF33, ART4, ASPA, AXIN2, B3GALT5-AS1, B4GALNT3, BAALC, BCL6B, BEX1, BICDL1, BLACAT1, BMP1, BTNL9, C10orf25, C10orf91, C12orf74, C19orf84, C1orf53, CA2, CADM3-AS1, CALCA, CALCRL, CASP12, CAV1, CCBE1, CCDC68, CCDC85A, CCM2L, CD24, CD300LG, CD36, CD93, CDH5, CDO1, CDT1, CEACAM6, CEACAM7, CHRNA4, CLDN18, CLEC1A, CLIC5, CLIC6, CNTLN, COL10A1, COL13A1, COL4A6, COL6A6, CORO6, CP, CYTH4, CYYR1, DACH1, DMRTC1B, DPP6, DSCC1, DST, DUOX1, ECSCR, EDNRB, EFCC1, EMCN, EML1, EMP2, EPAS1, EPB41L2, EPT1, ERG, ERICH4, EYA4, FABP4, FAM107A, FAM124B, FAM135B, FAM65A, FAM83A, FAM83H, FAT3, FBXO40, FCN3, FENDRR, FGD5, FGFR4, FHL1, FHL5, FLI1, FLJ34503, FMO2, FOXF1, FPR1, FRMD3, GALNT13, GBP4, GCG, GDF15, GHR, GIMAP1, GIMAP8, GJB2, GNGT1, GNLY, GPC3, GPM6A, GPR182, GPX3, GRIA1, GRK5, GRM8, GSTM5, GUCY1A2, GYG2, GZMB, HABP2, HBA2, HBB, HCN3, HEG1, HHIP, HIF3A, HMGCLL1, HOXA10-HOXA9, HSPB6, HSPC324, IGHA2, IGHV4-31, IGK, IGKC, IGLJ3, IGSF10, IL18RAP, IL1RL1, IL23A, IL7R, ILDR1, INSC, IQGAP3, ITGA8, JAM2, KANK3, KCNB1, KCNK3, KCNT2, KCTD16, KIAA0087, KIAA1462, KIAA1614, KIF18B, KIF26B, KL, KLF13, KLF15, KLF4, KLRC3, KLRD1, KLRF1, KRT6A, LAMA3, LAMA4, LAPTM4B, LCAL1, LCNL1, LDB2, LDHAL6A, LEPROT, LIMK1, LIN7A, LINC00052, LINC00312, LINC00472, LINC00643, LINC00842, LINC00845, LINC00961, LINC00968, LINC01021, LINC01123, LINC01545, LOC100506725, LOC100507487, LOC100996760, LOC101927699, LOC101928161, LOC101928307, LOC101928417, LOC101929398, LOC101929486, LOC101930541, LOC102725051, LOC105379426, LOC285147, LOC400568, LOC400794, LTBP4, LYVE1, MACF1, MAOB, MAPRE2, MBD5, MCEMP1, MCOLN3, MEIS1, MESDC1, MFAP4, MGST1, MIR1204, MME, MMP1, MMP7, MRAP, MSMB, MTO1, MUC3, MUC3B, MYCT1, MYOC, MYZAP, NAALAD2, NAV2-IT1, NDRG2, NDRG4, NECAB2, NECTIN4, NEGR1, NEK2, NFASC, NOTCH4, NOVA2, NPR1, NPSR1, NQO1, NR5A2, NRG3, NTNG1, NTNG2, NTRK2, OR5E1P, OR5P2, OR5P3, OR6W1P, P2RY1, PAPOLB, PCAT19, PCDH15, PDE5A, PDE8B, PDGFB, PDK4, PDLIM3, PECAM1, PGR, PIR-FIGF, PKHD1L1, PPBP, PPP1R14A, PRF1, PRKCB, PROM2, PRPH, PRX, PTPRB, PTPRS, QKI, RAMP3, RASEF, RASIP1, RBP2, RGCC, RHOJ, RNF152, ROBO4, RTKN2, RXFP1, RYR2, S100B, S1PR1, S1PR5, SCARA5, SCN10A, SCN7A, SCNN1A, SDPR, SEC14L1, SEMA3G, SEMA5A, SEMA6D, SERPIND1, SFTPC, SGCG, SGPP2, SH2D3C, SH3GL3, SHANK3, SHROOM4, SIX1, SLC14A1, SLC19A3, SLC5A9, SLC6A4, SLCO2A1, SLIT2, SLIT3, SMAD6, SMAD9, SMPDL3B, SOX17, SOX7, SPDEF, SPINK1, SPOCK2, SPTBN1, SRCIN1, SSTR1, STARD13, STARD13-AS, STX11, STXBP6, SULT1C4, SUSD4, SV2C, SVEP1, SYCP2L, SYN2, TAS1R1, TBX2, TBX3, TBX4, TCEAL2, TCF21, TEK, TFAP2A, TGFBR3, TIE1, TIMP3, TMC2, TMEM100, TMEM246, TMEM252, TMEM45B, TMEM47, TMEM74, TMEM74B, TMEM75, TMIE, TMPRSS4, TMTC1, TNNC1, TNR, TNXB, TOX3, TRDV3, TRHDE, TRHDE-AS1, TRIM58, TRPA1, TRPC6, TTC16, TTLL7, TTN, URB1, VGLL3, VIPR1, VWF, WFDC2, WISP2, WNT7B, ZNF366, ZNF471, ZNF662, ZNF677, ZPLD1
GSE136043	554	AATK, ABCA12, ABCB1, ABCC3, ABLIM3, ACADL, ACAN, ACAP3, ADAM8, ADAMTS14, ADAMTS16, ADAMTS8, ADAMTSL3, ADCY8, ADH1A, ADH1B, ADM2, ADORA3, ADRA1D, ADRB2, AGER, AGR2, AGTR1, AHNAK, AKNA, AKR1B10, ANKFN1, ANKRD18B, ANKRD20A2, ANKRD20A5P, ANKRD20A9P, ANKRD22, ANKRD29, ANXA10, ANXA3, AOC3, AOX1, APEH, APLN, AQP4, AQP6, ARHGAP6, ARHGEF16, ARHGEF19, ARHGEF26, ASPA, ASPHD1, ASS1, ATL2, ATP10B, B3GAT1, B3GNT3, BAI3, BCAS1, BCL6B, BDNF, BIK, BPIFA1, BPIFB1, BTNL8, BTNL9, C10orf116, C10orf140, C10orf67, C10orf81, C12orf39, C1orf81, C1QTNF4, C1QTNF7, C20orf202, C2CD4A, C4orf49, C6orf174, C6orf222, C8B, C9orf125, C9orf129, C9orf140, CA4, CADM3, CALCA, CAMK1D, CAMK2N1, CAPN13, CAPN8, CASP12, CASR, CAV1, CAV2, CAV3, CCDC129, CCDC48, CCDC85A, CCK, CCL20, CCL24, CCNO, CCRL1, CD180, CD24, CD36, CD79A, CDCA7, CDH13, CDH3, CDO1, CEACAM1, CEACAM3, CEACAM5, CEACAM6, CEACAM7, CES1, CFD, CHAC1, CHRDL1, CHRM1, CKMT1A, CLDN18, CLDN4, CLEC1B, CLEC3B, CLIC5, CLIC6, CNOT3, CNTN6, CNTNAP3, COL10A1, COL13A1, COL17A1, COL24A1, COL6A6, COMP, COPG2IT1, COX2, CP, CPB2, CPNE4, CPNE5, CPNE7, CST6, CT45A5, CXCL13, CXCL14, CYP1B1, CYP27B1, DACH1, DCDC2, DEFA3, DEFA4, DEPTOR, DERL3, DFNB31, DLX5, DMRT2, DNASE1L3, DPEP3, DPP6, DPT, DSP, E2F8, ECEL1P2, EDN3, EFNB3, EHF, ELF3, ELFN2, ELMOD1, EMCN, EMP2, EPHA10, EPN3, ERN2, ERV18-1, FAM105A, FAM150B, FAM155B, FAM162B, FAM189A2, FAM84A, FBLIM1, FBN3, FCGBP, FER1L4, FERMT2, FGD5, FGF12, FGF2, FGL1, FHL2, FIBIN, FIGF, FLJ13744, FLJ30901, FLJ34503, FLJ44635, FMO2, FOXF1, FOXF2, FRAS1, FRMD3, FRMD5, FUT1, FUT3, FUT8, FZD10, GAPDH, GCG, GDF10, GHR, GJB2, GJC2, GKN2, GMDS, GNG11, GNGT1, GOLGA7B, GOLM1, GPA33, GPC3, GPC5, GPIHBP1, GPM6A, GPR110, GPR158, GPR160, GPR4, GPT2, GREM1, GRIA1, GRIP1, GRK5, GRRP1, GSTA2, GSTA5, HAMP, HAS1, HCN4, HDGF, HES7, HHIP, HIGD1B, HMGB3, HNRNPH1, HOXA4, HOXA5, HS3ST1, HS6ST2, HSH2D, HSPA12B, IER3, IFITM1, IGFBP2, IGSF10, IGSF9, IHH, IL16, IL20RA, INHBB, INMT, ISYNA1, ITGA8, ITLN1, ITLN2, IYD, JAKMIP2, KAL1, KANK3, KANK4, KCNA4, KCNK12, KDELR3, KHDRBS2, KIAA1217, KIAA1324, KIAA1462, L1TD1, LAD1, LAMA3, LCN2, LDB2, LEMD1, LEPR, LGALS4, LGR4, LIN7A, LINC00163, LINC00472, LINGO1, LMF1, LOC100127983, LOC100128905, LOC100129463, LOC100130428, LOC100130899, LOC100131094, LOC100133669, LOC100192426, LOC100505933, LOC100507055, LOC158376, LOC283392, LOC284080, LOC338653, LOC388906, LOC389023, LOC389033, LOC389332, LOC400550, LOC400568, LOC553137, LOC572558, LOC643650, LOC643988, LOC645431, LOC646324, LOC646513, LOC729860, LOC731424, LOC91948, LPHN2, LPL, LRP4, LRRC15, LRRC19, LRRC31, LRRN3, LRRN4CL, LRRTM4, LTF, LUZP2, LYG2, LYPD5, MAGED4B, MAL, MAMDC2, MAMDC4, MANEAL, MAP7D2, MAPK4, MARCO, MDK, MEF2D, MESP1, MESP2, MFAP4, MFSD6L, MGAT3, MGC20647, MIOX, MME, MMP11, MMP12, MNX1, MOGAT1, MPP6, MS4A15, MSR1, MUC4, MX2, MYH2, MYOC, MYOM2, MYZAP, NCKAP5, NDNF, NDRG4, NECAB1, NGEF, NHS, NOS1AP, NOS2, NOV, NPSR1, NR5A2, NRG1, NTNG1, OCIAD2, ODAM, OR5L2, OTX1, OVOL1, P2RY1, PAX9, PCA3, PCDH9, PCM1, PCOLCE2, PDE1C, PDE5A, PDE6A, PEAR1, PHLDA2, PIN1P1, PIP5K1B, PITPNM2, PITX1, PITX2, PKHD1L1, PKIB, PKNOX2, PLAC9, PLEKHH2, PLIN5, PNMA6C, POPDC3, POU3F2, PPARGC1A, PPDPF, PPP1R17, PRKG2, PROM1, PROS1, PRSS50, PSAPL1, PSAT1, PTN, PTPRD, PTPRZ1, PVRL4, PYCR1, QRFPR, RAB26, RADIL, RAG1, RALGPS2, RAMP1, RANBP3L, RAPH1, RASIP1, RBP2, RECK, REEP6, RET, RETN, RGS17, RGS9BP, RHBDL2, RN28S1, RNASE13, RNF122, RNF182, ROBO4, RPL28, RSPO1, RSPO2, RTKN2, RUNDC3B, RUNX1, S100A3, S100B, SAA1, SAA2, SAA4, SALL4, SBK1, SCD5, SCEL, SCN3B, SCN4B, SCN7A, SEMA3B, SEMA3D, SEMA3E, SEMA5A, SERTM1, SGCG, SGPP2, SH2D1B, SH3GL2, SH3GL3, SIDT1, SIX1, SIX4, SLC15A1, SLC16A12, SLC19A3, SLC1A1, SLC1A7, SLC22A18, SLC22A18AS, SLC2A5, SLC39A8, SLC44A4, SLC46A2, SLC7A5, SLC8A2, SLCO4C1, SLIT2, SMOX, SNCB, SOSTDC1, SOX2, SOX7, SPINK1, SPOCK2, SPP1, SPRYD7, SPTBN1, SPTBN2, SRRM4, SSTR1, ST6GALNAC1, ST6GALNAC5, STEAP3, STRA6, STXBP6, SUN1, SUSD4, SVEP1, SYNM, SYNPO2L, SYT12, TAL1, TBX3, TBX4, TCEA2, TCF21, TEK, TFF3, TGM1, TINAGL1, TMEM100, TMEM132C, TMEM139, TMEM238, TMEM63A, TMEM63C, TMPRSS4, TNNC1, TNXB, TOX3, TSPAN1, TTC39A, TTI2, TTN, TUSC1, UACA, UNC5CL, USHBP1, VEGFA, VGLL3, VPREB3, VSTM2L, VTCN1, VWF, WFDC2, WIF1, WNK2, WNT3A, ZBED2, ZNF331, ZNF365, ZNF534, ZNF626
GSE140797	641	ABCA12, ABCA4, ABCA6, ABCA8, ABCB4, ABCC3, ABLIM3, ACACB, ACADL, ACOXL, ACTG2, ACVRL1, ADAMDEC1, ADAMTS14, ADAMTS8, ADARB1, ADCY8, ADH1A, ADH1B, ADM2, ADRB1, ADRB2, AGER, AGR2, AGTR1, AIM2, AK4, AKNAD1, AKR1B10, AKR1B15, ANGPT1, ANGPTL1, ANGPTL7, ANKRD20A2, ANKRD20A9P, ANKRD29, ANKRD30BP2, ANKRD34B, ANLN, ANXA8L2, AOC3, APOE, AQP4, ARAP3, ARHGAP29, ARHGAP6, ARHGEF16, ARHGEF4, ASPA, ASPM, ATP10B, ATP1A2, AURKAPS1, B3GNT3, BAALC, BCHE, BEX1, BIRC5, BMPER, BMPR2, BNIP2, BPIFA1, BRIP1, BTNL3, BTNL9, BUB1, C10orf116, C10orf67, C10orf81, C11orf88, C12orf39, C14orf132, C15orf48, C18orf56, C1orf173, C1orf194, C1orf81, C20orf160, C2orf40, C4orf7, C5orf4, C6orf174, C6orf222, C6orf225, C8B, C9orf125, C9orf85, CA4, CABYR, CACNA2D2, CADM1, CALCA, CALCRL, CAMK2N1, CAMP, CASQ2, CAV1, CAV2, CAV3, CBFA2T3, CCDC129, CCDC147, CCDC48, CCDC68, CCDC85A, CCL14, CCL19, CCL23, CCL7, CCNB2, CCNE1, CCNO, CD24, CD36, CDC45, CDCA2, CDCA3, CDCA5, CDCA7, CDH13, CDH19, CDHR4, CDKN2A, CDKN3, CDO1, CDT1, CEACAM3, CEACAM5, CENPA, CENPF, CENPM, CEP55, CFD, CGNL1, CHI3L1, CHIA, CHRDL1, CHRM1, CILP, CIT, CKM, CLDN18, CLDN3, CLEC1B, CLEC3B, CLIC5, CNTNAP2, COL10A1, COL13A1, COL17A1, COL1A1, COL28A1, COL3A1, COL4A3, COL6A6, COMP, COPG2IT1, COX7A1, CP, CPB2, CR2, CRABP2, CRTAC1, CTHRC1, CTLA4, CX3CR1, CXCL10, CXCL13, CXCL14, CXCL9, CXCR5, CXorf41, CXorf61, CYP3A5, CYP4B1, CYS1, CYYR1, DACH1, DACT2, DCDC2B, DES, DFNB31, DIXDC1, DLGAP5, DLX5, DMRT2, DMRTA2, DNAH12, DNASE1L3, DPP6, DUOX1, DUSP10, E2F8, ECEL1P2, ECSCR, EDN2, EDNRB, EEF1A2, EFCAB1, EGFL7, ELF3, EMCN, EMP2, ENPP3, EPAS1, EPHA10, ERCC6L, ERN2, ESCO2, ETS2, ETV1, EXO1, EZH2, F11, FABP4, FAM105A, FAM107A, FAM13C, FAM150B, FAM162B, FAM167A, FAM189A2, FAM3D, FANCA, FAP, FBLIM1, FCN3, FCRL4, FCRL5, FER1L4, FEZ1, FGF2, FHL1, FIBIN, FIGF, FLJ13744, FLJ30901, FLJ34503, FMO2, FOXF1, FRMD3, FRMD5, FRY, FUT3, FZD4, GAD1, GALNTL4, GAPDH, GCG, GDA, GDF10, GDF15, GENE_SYMBOL, GFOD1, GIMAP8, GINS1, GJB2, GJC2, GKN2, GLDN, GNAZ, GNG11, GNGT1, GOLGA7B, GPC3, GPER, GPIHBP1, GPM6A, GPM6B, GPR110, GPR133, GPRIN2, GPX3, GRASP, GREM1, GRK5, GRRP1, GSDMC, GUCY1B2, HAMP, HBA2, HBB, HBD, HBG1, HCAR3, HECW2, HEG1, HHIP, HIGD1B, HIST1H2AI, HJURP, HLF, HMGA2, HMGB3, HORMAD1, HPGD, HRASLS2, HS6ST2, HSD17B6, HSPA12B, HSPB2, IGF2BP3, IGFBP1, IGFBP3, IGFL2, IGLL1, IGLL5, IGSF10, IGSF9, IL18R1, IL1RL1, IL2RA, IL4I1, IL7R, INHBB, INMT, IRX1, IRX2, ITGA10, ITGA8, ITLN1, ITLN2, JAM2, JPH4, KAL1, KANK3, KANK4, KCNB1, KCNN4, KDELR2, KHDRBS2, KIAA0408, KIAA1324L, KIAA1462, KIF18A, KIF23, KISS1R, KLRD1, KRT14, KRT15, KRT17, KRT23, KRT8, KRT80, KRT8P12, L1TD1, LAD1, LAMA3, LAMP3, LCN2, LDB2, LEMD1, LEPR, LGALS4, LGI2, LGSN, LHFP, LIFR, LILRB4, LIMCH1, LIMS2, LINC00261, LINC00312, LINGO1, LOC100127983, LOC100129940, LOC100131395, LOC254896, LOC283392, LOC284080, LOC284276, LOC389023, LOC400550, LOC400568, LOC400891, LOC643037, LOC645431, LOC729860, LPL, LRRC10B, LRRC36, LRRK2, LRRN3, LTF, LY6K, LYPD1, LYVE1, MAGEA6, MAMDC2, MAOA, MAP3K15, MAPK4, MARCO, MATN3, MDK, MELK, MEX3A, MFAP4, MGAT3, MIOX, MIR205HG, MLF1IP, MMP1, MMP11, MMP12, MMP13, MMP7, MMP9, MMRN1, MND1, MNX1, MOGAT1, MS4A15, MSRB3, MUC16, MUC21, MUC4, MYH10, MYH11, MYH2, MYOC, MYOM2, MYZAP, MZB1, NCKAP5, NDC80, NECAB1, NEDD4L, NEIL3, NEK2, NEXN, NMNAT2, NOSTRIN, NOTCH4, NOTUM, NPNT, NPR3, NPSR1, NPY1R, NTNG1, NTRK3, NUF2, NUSAP1, NXPH4, OCA2, ODZ2, OGN, PABPC1L, PADI1, PAQR5, PAX9, PBK, PCDH7, PCOLCE2, PCSK9, PDE1C, PDE5A, PDE8B, PDK4, PDZD2, PDZRN4, PEBP4, PECAM1, PHLDA2, PI16, PIK3R1, PITX1, PITX2, PKHD1L1, PLA2G1B, PLAC9, PLAU, PLCXD3, PLEKHH2, POU2AF1, PP14571, PPP1R3C, PRKCE, PRSS22, PSCA, PTCH1, PTGFR, PTPN21, PTPRB, PTPRM, PTPRQ, PVRL4, PYCR1, RAB26, RAD54L, RADIL, RALGPS2, RASIP1, RBP2, RECK, RECQL4, REG1A, RETN, RGS1, RHBDL2, RNF182, RNFT2, ROBO4, RP11-165H20.1, RSPO2, RTKN2, RUNX1, RXFP1, S100A2, S100P, S1PR1, SALL4, SCARA5, SCN1A, SCN4B, SCN7A, SDPR, SEC14L3, SEC14L4, SELENBP1, SEMA3E, SEMA3G, SEMA5A, SEPP1, SFN, SFTPD, SGCA, SGCG, SGPP2, SH2D3C, SHANK3, SIX1, SIX4, SLC14A1, SLC16A12, SLC17A9, SLC1A1, SLC2A1, SLC39A8, SLC5A8, SLC6A4, SLC7A11, SLCO2A1, SLIT2, SLIT3, SLITRK3, SMAD6, SMAD9, SNX1, SNX22, SOSTDC1, SPAG5, SPARCL1, SPINK1, SPOCK2, SPOCK3, SPP1, SPRYD7, SSTR1, ST6GALNAC1, STAC, STARD9, STEAP1, STIL, STRA6, STX1A, STYK1, SULF1, SULT1C2, SUSD2, SVEP1, SYCP3, SYN2, SYNE1, SYNM, SYNPO2, SYNPO2L, SYT12, TAL1, TBC1D3B, TBC1D3G, TBX2, TBX3, TCAM1P, TCF21, TCN1, TDO2, TDRD1, TEK, TEKT5, THBD, THBS2, THSD4, THY1, TIMP1, TIMP3, TK1, TMEM100, TMEM45B, TMEM47, TMEM63C, TMPRSS4, TMSB15A, TNS4, TNXB, TOP2A, TPPP, TSPAN12, TTK, TTN, TUBB1, TUBB2B, TUBB3, TWIST1, TYMS, UBD, UBE2C, UBE2T, UCK2, UHRF1, VAPA, VEPH1, VGF, VGLL3, VIL1, VIPR1, VMP1, VSTM2L, WDR16, WDR17, WFDC2, WIF1, WISP2, WNT3A, XAGE1A, XDH, ZBED2, ZNF300P1, ZNF385B, ZWINT, ZYG11A

**Table 6:** GO analysis and KEGG pathway analysis of 77 common DEGs in 3 GEO datasets

Category	GO ID	Term	Count	*P* value
BP	GO:0007155	Cell adhesion	12	2.86E-06
BP	GO:0030198	Extracellular matrix organization	6	0.001
BP	GO:0001525	Angiogenesis	6	0.002
BP	GO:0007267	Cell–cell signaling	6	0.004
BP	GO:0016337	Single organismal cell–cell adhesion	5	8.34E-04
BP	GO:0001570	Vasculogenesis	4	0.002
BP	GO:0001666	Response to hypoxia	4	0.035
BP	GO:0001763	Morphogenesis of a branching structure	3	4.72E-04
BP	GO:0001958	Endochondral ossification	3	0.005
BP	GO:0007188	Adenylate cyclase-modulating G-protein coupled receptor signaling pathway	3	0.010
BP	GO:0010811	Positive regulation of cell–substrate adhesion	3	0.011
BP	GO:0001657	Ureteric bud development	3	0.011
BP	GO:0007190	Activation of adenylate cyclase activity	3	0.012
BP	GO:0001658	Branching involved in ureteric bud morphogenesis	3	0.013
BP	GO:0031623	Receptor internalization	3	0.014
BP	GO:0007189	Adenylate cyclase-activating G-protein coupled receptor signaling pathway	3	0.018
BP	GO:0008104	Protein localization	3	0.027
BP	GO:0030574	Collagen catabolic process	3	0.029
BP	GO:0043627	Response to estrogen	3	0.030
BP	GO:0030334	Regulation of cell migration	3	0.038
BP	GO:0051897	Positive regulation of protein kinase B signaling	3	0.048
BP	GO:0045986	Negative regulation of smooth muscle contraction	2	0.017
BP	GO:0070836	Caveola assembly	2	0.021
BP	GO:0043549	Regulation of kinase activity	2	0.021
BP	GO:0060426	Lung vasculature development	2	0.025
BP	GO:0048251	Elastic fiber assembly	2	0.029
BP	GO:0002024	Diet-induced thermogenesis	2	0.037
BP	GO:0030857	Negative regulation of epithelial cell differentiation	2	0.045
BP	GO:0045779	Negative regulation of bone resorption	2	0.049
BP	GO:0001944	Vasculature development	2	0.049
CC	GO:0005886	Plasma membrane	28	0.0029
CC	GO:0005576	Extracellular region	17	3.80E-04
CC	GO:0005615	Extracellular space	12	0.015
CC	GO:0005887	Integral component of plasma membrane	11	0.047
CC	GO:0009986	Cell surface	8	0.005
CC	GO:0005578	Proteinaceous extracellular matrix	7	6.16E-04
CC	GO:0045121	Membrane raft	5	0.009
CC	GO:0005581	Collagen trimer	4	0.006
CC	GO:0005667	Transcription factor complex	4	0.040
CC	GO:0045177	Apical part of cell	3	0.035
MF	GO:0008201	Heparin binding	4	0.024
KEGG	hsa04151	PI3K-Akt signaling pathway	6	0.044
KEGG	hsa04512	ECM–receptor interaction	5	0.001
KEGG	hsa04510	Focal adhesion	5	0.028
KEGG	hsa05414	Dilated cardiomyopathy	4	0.012

### Potential molecular mechanism of the 6 hub genes in LUAD

**Figure 5:**
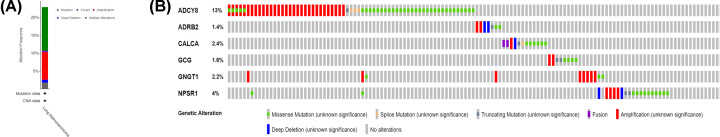
The genetic alterations of 6 hub genes in LUAD (**A**) The alteration frequency of 6 hub genes. (**B**) Mutations of every hub gene. Green represented missense mutation, orange represented splice mutation, dark grey represented truncating mutation, violet represented fusion, red represented amplification, and blue represented deep deletion.

We analyzed the alterations of 6 hub genes by using the cBioPortal online tool for TCGA LUAD cohort. The mutation, which could change the function of protein by changing gene sequence, was the most common gene alteration in all hub genes (Figure 5A). In the present study, the OncoPrint from cBioPortal showed that 24% (141/580) cases with genetic alterations could be obtained, the ADCY8, ADRB2, CALCA, GCG, GNGT1, and NPSR1 had genetic alterations, including missense mutation, splice mutation, truncating mutation, fusion, amplification, and deep deletion (Figure 5B). The ADCY8 (13%) was the most frequently altered gene among the 6 hub genes, including missense mutation, splice mutation, truncating mutation, and amplification.

### Transcription levels of 6 hub genes in LUAD tissues and normal lung tissues

**Figure 6:**
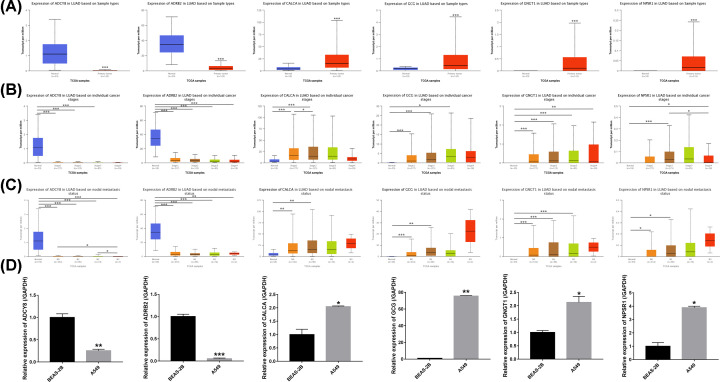
Relative expression and the correlation between 6 hub genes in LUAD (**A**) The expression of 6 hub genes in LUAD patients (Ualcan database). (**B**) Correlation between expression of 6 hub genes and tumor stages in LUAD patients (Ualcan database). (**C**) Expression of 6 hub genes in LUAD based on nodal metastasis status (UALCAN database). (**D**) Quantitative real-time PCR results for the 6 hub genes. Expression of hub genes were normalized against GAPDH expression. The statistical significance of differences was calculated by the Student’s *t-*test; *P<0.05, **P<0.01, ***P<0.001.

The online database UALCAN was used to analyze the expression of the 6 hub genes in LUAD tissues and normal lung tissues. The results showed that compared with normal lung tissues, the expression levels of ADCY8 and ADRB2 were down-regulated and the expression levels of CALCA, GCG, GNGT1, and NPSR1 were up-regulated in LUAD tissues (all *P*<0.001) (Figure 6A). In terms of the TNM stage, the ADCY8 expression level in normal lung tissues was higher than in stage I, stage II, and stage III tissues (all *P*<0.001), the ADRB2 expression level in normal lung tissues was higher than in stage I, stage II, stage III, and stage IV tissues (all *P*<0.001), the CALCA expression level in normal lung tissues was lower than in stage I and stage II tissues (all *P*<0.001), the GCG expression level in normal lung tissues was lower than in stage I, stage II, and stage III tissues (*P*<0.001, *P*<0.001, *P*=0.038, respectively), the GNGT1 expression level in normal lung tissues was higher than in stage I, stage II, stage III, and stage IV tissues (*P*<0.001, *P*<0.001, *P*<0.001, *P*=0.001, respectively), and the NPSR1 expression level in normal lung tissues was higher than in stage II and stage IV tissues (*P*<0.001, *P*=0.011, respectively) (Figure 6B). In terms of the nodal metastasis, the ADCY8 expression level in normal lung tissues was higher than in N0, N1, N2, and N3 tissues (all *P*<0.001), the ADRB2 expression level in normal lung tissues was higher than in N0, N1, N2, and N3 tissues (*P*<0.001, *P*<0.001, *P*<0.001, *P*=0.006, respectively), the CALCA expression level in normal lung tissues was lower than in N0 and N1 tissues (all *P*<0.001), the GCG expression level in normal lung tissues was lower than in N0 and N1 tissues (*P*<0.001, *P*=0.005, respectively), the GNGT1 expression level in normal lung tissues was higher than in N0, N1, and N2 tissues (all *P*<0.001), the NPSR1 expression level in normal lung tissues was lower than in N0 and N1 tissues (*P*=0.013, *P*=0.021, respectively) (Figure 6C).

To further verify the results of bioinformatics analysis, the mRNA levels of the 6 hub genes were determined in Beas-2B cells and A549 cells with qRT-PCR. As illustrated in Figure 6D, the ADCY8 and ADRB2 were significantly down-regulated in A549 cells compared with Beas-2B cells (all *P*<0.05), while the CALCA, GCG, GNGT1, and NPSR1 were signficantly up-regulated in A549 cells compared with Beas-2B cells (all *P*<0.05), as predicted by the bioinformatics analysis.

### The protein expression levels of 6 hub genes in LUAD tissues

**Figure 7:**
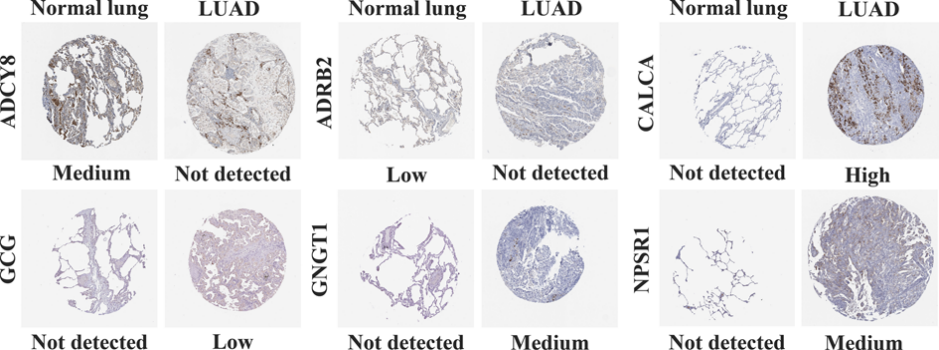
The IHC - based protein expression of 6 hub genes in LUAD tissues and normal lung tissues All the IHC staining images were obtained from the HPA database.

To determine the differentially protein expression of 6 hub genes in LUAD, IHC staining images for the hub genes proteins in LUAD tissues as well as normal lung tissues were obtained from the HPA database. Consistent with the above results, the results showed that the protein expression levels of CALCA, GCG, GNGT1, and NPSR1 were higher in LUAD tissues than that in normal lung tissues, while the protein expression levels of ADCY8 and ADRB2 were lower in LUAD tissues than that in normal lung tissues (Figure 7).

### Relationship between the 6 hub genes and prognosis of LUAD patients

**Figure 8:**
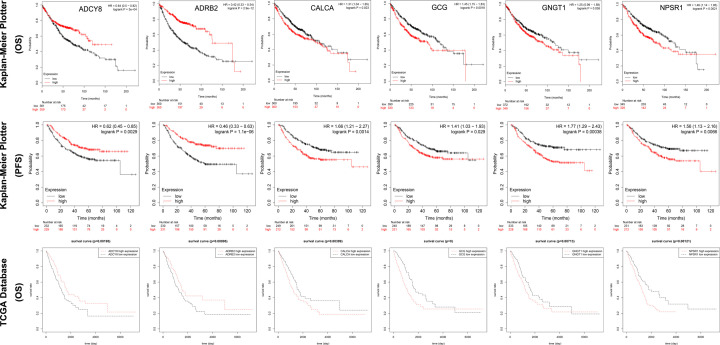
The relationship of prognosis and expression level of 6 hub genes in patients with LUAD The prognostic significance of the 6 hub genes in patients with LUAD, according to the Kaplan–Meier plotter database and TCGA database. The red curve represented the survival curve of LUAD patients with high gene expression, the black curve represented the survival curve of LUAD patients with low gene expression.

The overall survival (OS) and progression-free survival (PFS) were analyzed for patients with LUAD using the Kaplan–Meier survival plot. Briefly, the 6 genes were uploaded to the database and Kaplan–Meier curves were plotted. The results indicated that low expression of ADCY8 [HR = 0.64 (0.50–0.82), *P*=3e-04] and ADRB2 [HR = 0.42 (0.33–0.54), *P*=2.9e-12], and high expression of CALCA [HR = 1.31 (1.04–1.65), *P*=0.023], GCG [HR = 1.45 (1.15–1.83), *P*=0.0018], GNGT1 [HR = 1.25 (0.99–1.58), *P*=0.038], and NPSR1 [HR = 1.46 (1.14–1.85), *P*=0.0021] were correlated with significantly poor OS in LUAD patients (Figure 8). Moreover, low expression of ADCY8 [HR = 0.62 (0.45–0.85), *P*=0.0029] and ADRB2 [HR = 0.46 (0.33–0.63), *P=*1.1e-06], and high expression of CALCA [HR = 1.66 (1.21–2.27), *P*=0.0014], GCG [HR = 1.41 (1.03–1.93), *P*=0.029], GNGT1 [HR = 1.77 (1.29–2.43), *P*=0.00038], and NPSR1 [HR = 1.56 (1.13–2.16), *P*=0.0066] were correlated with significantly poor PFS in LUAD patients (Figure 8).

Subsequently, we downloaded the survival data of patients with LUAD from TCGA database, combined the survival data and gene expression data, and divided them into high expression group and low expression group to study the effect of 6 hub genes on the prognosis of patients with LUAD, the results showed that high expression of ADRB2 (*P*=0.00895) and ADCY8 (*P*=0.00195), and lower expression of CALCA (*P*=0.00399), GCG (*P*=0), GNGT1 (*P*=0.00713), and NPSR1 (*P*=0.00121) were correlated with significantly better OS in LUAD patients (Figure 8), which were consistent with the results of Kaplan–Meier survival plot.

### ROC of the 6 hub genes in patients with LUAD

**Figure 9:**
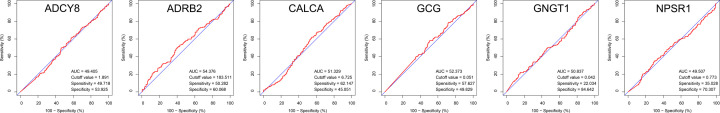
ROC curves for the 6 hub genes in LUAD Red represented sensitive curve, blue indicated identify line. The *X* axis showed false positive rate, presented as ‘100-Specificity (%)’. The *Y* axis indicated true positive rate, shown as ‘Sensitivity (%)’.

We obtained the gene expression data and clinical data from TCGA database. ROC curve analysis was performed in R software using procedures from the ‘pROC’ package. The results of the ROC curves indicated that the 6 hub genes had different specificity and sensitivity in predicting the OS of LUAD patients (Figure 9). However, all the area under receiver operating characteristics (AUCs) were lower than 55, which indicated that the predictive role of 6 hub genes were poor.

### Relationship between the 6 hub genes and clinical features in LUAD patients

There were 489 LUAD patients in TCGA database, including 470 patients with age data, 233 patients ≥65 years old and 237 patients <65 years old. There were 489 patients with gender data, 223 men and 266 women. There were 486 patients with data of primary tumor, 426 patients in stage T1-2 and 60 patients in stage T3-4. There were 475 patients with data of nodal metastasis, 317 patients without nodal metastasis and 158 patients with nodal metastasis. There were 357 patients with data of distant metastasis, 333 patients without distant metastasis and 24 patients with distant metastasis. There were 481 patients with data of TNM stage, 378 patients in stage I-II and 103 patients in stage III-IV (Table 7).

**Table 7:** The clinicopathologic features of LUAD patients in TCGA database

Clinical characteristics	Subgroup	Total (*N*)	Expression of ADCY8		Expression of ADRB2		Expression of CALCA		Expression of GCG		Expression of GNGT1		Expression of NPSR1	
			Low	High	Low	High	Low	High	Low	High	Low	High	Low	High
Age	≤65	233	172	61	119	114	119	114	102	131	117	116	173	60
	>65	237	162	75	115	122	115	122	132	105	117	120	161	76
Gender	male	223	102	121	126	97	105	118	121	102	111	112	108	115
	female	266	141	125	118	148	265	1	122	144	132	134	135	131
Primary tumor	T1-2	426	185	241	181	245	228	198	222	204	221	205	227	199
	T3-4	60	41	19	50	10	24	36	30	30	21	39	20	40
Nodal metastasis	N0	317	152	165	141	176	223	94	180	157	186	121	177	140
	N1-3	158	89	69	96	62	44	114	37	121	51	107	60	98
Distant metastasis	M0	333	144	189	135	198	193	140	188	145	173	160	184	149
	M1	24	19	5	20	4	6	18	7	17	9	15	11	13
Stage	I-II	378	177	201	163	215	208	170	201	177	197	181	204	174
	III-IV	103	63	40	76	27	42	61	39	64	43	60	36	67

**Figure 10:**
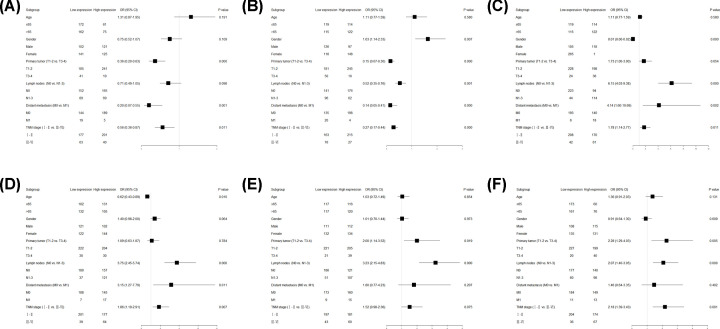
Forest plots of OR for the association between 6 hub genes expression and clinicopathological features in patients with LUAD (**A**) ADCY8, (**B**) ADRB2, (**C**) CALCA, (**D**) GCG, (**E**) GNGT1, (**F**) NPSR1.

Pearson’s chi-squared test showed that the expression level of ADCY8 was negatively correlated with T stage [OR = 0.36 (0.20–0.63), *P*=0.000], distant metastasis [OR = 0.20 (0.07–0.55), *P*=0.001], and TNM stage [OR = 0.56 (0.36–0.87), *P*=0.011]. The expression level of ADRB2 in female was significantly higher than in male [OR = 1.63 (1.14–2.33), *P*=0.007], and the expression level of ADRB2 was negatively correlated with T stage [0.15 (0.07–0.30), *P*=0.000], nodal metastasis [OR = 0.52 (0.35–0.76), *P*=0.001], distant metastasis [OR = 0.14 (0.05–0.41), *P*=0.000], and TNM stage [OR = 0.27 (0.17–0.44), *P*=0.000]. The expression level of CALCA in male was significantly lower than in female [OR = 0.01 (0.00–0.02), *P*=0.000], and the expression level of CALCA was positively correlated with nodal metastasis [OR = 6.15 (4.03–9.38), *P*=0.000], distant metastasis [OR = 4.14 (1.60–10.69), *P*=0.002], and TNM stage [OR = 1.78 (1.14–2.77), *P*=0.011]. The expression level of GCG was negatively correlated with age [OR = 0.01 (0.00–0.02), *P*=0.010], and the expression level of GCG was positively correlated with nodal metastasis [OR = 3.75 (2.45–5.74), *P*=0.000], distant metastasis [OR = 3.15 (1.27–7.79), *P*=0.011], and TNM stage [OR = 1.86 (1.19–2.91), *P*=0.007]. The expression level of GNGT1 was positively correlated with T stage [OR = 2.00 (1.14–3.52), *P*=0.019] and nodal metastasis [OR = 3.23 (2.15–4.83), *P*=0.000]. The expression level of NPSR1 was positively correlated with T stage [OR = 2.28 (1.29–4.03), *P*=0.005], nodal metastasis [OR = 2.07 (1.40–3.05), *P*=0.000], and TNM stage [OR = 2.18 (1.39–3.43), *P*=0.001] (Figure 10, Table 8).

**Table 8:** Correlation between 6 hub genes expression and the clinicopathologic features of LUAD patients

Clinical characteristics	Total (*N*)	OR in ADCY8 expression	*P* value	OR in ADRB2 expression	*P* value	OR in CALCA expression	*P* value
Age (≥65 vs. <65)	470	1.31 (0.87–1.95)	0.191	1.11 (0.77–1.59)	0.580	1.11 (0.77–1.59)	0.580
Gender (male vs. female)	489	0.75 (0.52–1.07)	0.109	**1.63 (1.14–2.33)**	**0.007**	**0.01 (0.00–0.02)**	**0.000**
Primary tumor (T1-2 vs. T3-4)	486	**0.36 (0.20–0.63)**	**0.000**	**0.15 (0.07–0.30)**	**0.000**	1.73 (1.00–3.00)	0.054
Nodal metastasis (N0 vs. N1-3)	475	0.71 (0.49–1.05)	0.098	**0.52 (0.35–0.76)**	**0.001**	**6.15 (4.03–9.38)**	**0.000**
Distant metastasis (M0 vs. M1)	357	**0.20 (0.07–0.55)**	**0.001**	**0.14 (0.05–0.41)**	**0.000**	**4.14 (1.60–10.69)**	**0.002**
Stage (I-II vs. III-VI)	481	**0.56 (0.36–0.87)**	**0.011**	**0.27 (0.17–0.44)**	**0.000**	**1.78 (1.14–2.77)**	**0.011**

		OR in GCG expression	*P* value	OR in GNGT1 expression	*P* value	OR in NPSR1 expression	*P* value
Age (≥65 vs. <65)	470	**0.62 (0.43–0.89)**	**0.010**	1.03 (0.72–1.49)	0.854	1.36 (0.91–2.03)	0.131
Gender (male vs. female)	489	1.40 (0.98–2.00)	0.064	1.01 (0.70–1.44)	0.973	0.91 (0.64–1.30)	0.609
Primary tumor (T1-2 vs. T3-4)	486	1.09 (0.63–1.87)	0.784	**2.00 (1.14–3.52)**	**0.019**	**2.28 (1.29–4.03)**	**0.005**
Nodal metastasis (N0 vs. N1-3)	475	**3.75 (2.45–5.74)**	**0.000**	**3.23 (2.15–4.83)**	**0.000**	**2.07 (1.40–3.05)**	**0.000**
Distant metastasis (M0 vs. M1)	357	**3.15 (1.27–7.79)**	**0.011**	1.80 (0.77–4.23)	0.207	1.46 (0.64–3.35)	0.402
Stage (I-II vs. III-VI)	481	**1.86 (1.19–2.91)**	**0.007**	1.52 (0.98–2.36)	0.075	**2.18 (1.39–3.43)**	**0.001**

## Discussion

LUAD is one of the important subtype of NSCLC with high morbidity and mortality [3]. LUAD is a product of cumulative genetic, epigenetic, somatic, and endocrine aberrations, therefore, understanding the biological mechanism of LUAD is of critical importance for clinical diagnosis and treatment. As microarrays have a wide range of applications in oncology, including identification of disease-associated biomarkers, alternative splicing, and gene function prediction, it has been widely used to predict the potential therapeutic targets for multiple cancers. In the present study, we extract the microarray data from TCGA, 5208 up-regulated and 722 down-regulated DEGs between LUAD samples and normal samples were identified using bioinformatics analysis. We selected the top 300 up-regulated and 300 down-regulated genes for our study. In order to obtain additional analysis of these DEGs, GO and KEGG analyses were performed using DAVID software.

The GO analysis results indicated that the up-regulated DEGs were primarily associated with nucleosome assembly, telomere organization, cellular protein metabolic process, extracellular region, nucleosome, nuclear chromosome, sequence-specific DNA binding, protein heterodimerization activity, and hormone activity, while the down-regulated DEGs were primarily enriched in angiogenesis, receptor internalization, cell surface receptor signaling pathway, plasma membrane, extracellular region, integral component of plasma membrane, heparin binding, ion channel binding, and receptor activity. Summarily, most of the functional enrichment was related to the structure and function of chromosomes. As we all know, chromosomal instability is a major form of genomic volatility and contributes to abnormal chromosomal structure and numbers. Micro-satellite instability and increased frequency of base-pair mutations are other described forms of genomic instability, and the genome instability that is a hallmark of cancer almost certainly contributes to further alter sequences in regulatory regions that can promote tumor progression [25]. Furthermore, the enriched KEGG pathways of up-regulated DEGs included transcriptional misregulation in cancer, viral carcinogenesis, and glucagon signaling pathway. Previous studies have highlighted the link between disease-associated transcriptional misregulation and various cancers, such as lung cancer [26], breast cancer [27], colorectal cancer [28], and renal cancer [29]. Oliveira et. al [30] found that many DNA viruses targeted multiple cellular pathways to support malignant transformation and tumor development. Down-regulated DEGs were related to cAMP signaling pathway, calcium signaling pathway, and ECM–receptor interaction. The cyclic AMP (cAMP) signaling pathway is activated by cAMP. The increase in cAMP levels activates target molecules, such as cAMP-dependent protein kinase, exchange protein directly activated by cAMP, and cyclic nucleotide-gated ion channels [31]. These target effector molecules regulate various cellular responses, including metabolism, gene expression, proliferation, and apoptosis. Therefore, cAMP signaling has been studied as a target for various disease treatments, including cancer [32]. Various alterations to key molecules of the cAMP signaling pathway have been observed in lung cancer, and phosphodiesterase inhibitors have been shown to synergize with cisplatin to induce apoptosis in a broad panel of human lung cancer cell lines. These findings present cAMP signaling as a promising cellular target for antitumor treatments [33,34]. Calcium is one of the small signaling molecules regulating various biological functions in cells. Cell cycle regulation and cell death have been suggested to closely correlate with the intracellular calcium ion ([Ca2+]i) concentration [35]. Under pathological conditions, such as ischemia–reperfusion injury and oxidative stress, the [Ca2+]i level has been reported to markedly increase in many types of cells. This condition is known as calcium overload, which eventually leads to the activation of pro-apoptotic factors, resulting in apoptosis [36]. Logan et al. [37] reported that pathological Ca^2+^ overload triggers cell death. ECM–receptor interaction pathways are the most up-regulated gene-enriched signaling pathways, which play an important role in the process of tumor shedding, adhesion, degradation, movement, and hyperplasia. The roles of ECM in other cancers have been proved. Previous studies reported that the ECM was up-regulated in prostate cancer tissue [38] and participated in the process of tumor invasion and metastasis in gastric cancer [39]. In addition, the ECM in colorectal cancer could promote the development of epithelial–mesenchymal transition (EMT) in cancer cells [40].

By constructing a PPI network with DEGs, our study identified the top 10 degree hub genes, including ADCY8, ADRB2, ALB, CALCA, F2, GCG, GNGT1, INS, NPSR1, and SST. Then, a significant module was subsequently constructed with 14 nodes, which gained the highest MCODE score. After combining the results of MCODE and CytoHubba, 6 hub genes were chosen, including ADCY8, ADRB2, CALCA, GCG, GNGT1, and NPSR1. ADCY8 is a member of the adenylyl cyclase family of genes and produces the enzyme adenylyl cyclase (AC8). Orchel et al. [41] found that ADCY8 cause disturbance in the underlying biological processes, which could be important for the pathogenesis of endometrial cancer. ADRB2, located on chromosome 5q31-q32, consists of a single exon of 2,015 nucleotides, encoding a 413 amino acid protein for the beta-2-adrenergic receptor. The beta-adrenergic receptor is a member of the G-protein-coupled adrenergic receptor family and functions in adipose tissue by stimulating lipolysis, which affects lipid mobilization within human fat cells and the regulation of energy expenditure [42]. To date, only one epidemiologic studies have examined the association of genetic variation in ADRB2 with breast cancer risk among postmenopausal breast cancer [43]. The CALCA gene codes for calcitonin, an important regulator of bone calcium metabolism. Previous studies have reported that CALCA is a candidate gene for tumor-specific hypermethylation in cancer [44–46]. The expression level of CALCA protein is related to the pathological process of cervical cancer [47], and the methylation of CALCA gene promoter is closely related to the occurrence of cervical cancer [48]. The proglucagon gene (GCG), located on chromosome 2q24.2, can be activated by TCF7L2 in the Wnt signaling pathway, and expresses glucagon-like peptide1 (GLP-1) in the intestine [49]. GLP-1 plays an essential role in regulating blood glucose level by stimulating glucose-dependent insulin secretion [50]. A previous study suggested that higher incidences of pancreatic and medullary thyroid carcinoma in patients treated with GLP1 agonists [51]. GNGT1 located on chromosome 7q21.3 and code the gamma subunits of transducin [52]. Transducin, also known as GMPase, mediates the activation of a cyclic GTP-specific (guanosine monophosphate) phosphodiesterase by rhodopsin. Zhang et al. [53] reported that the expression level of GNGT1 in NSCLC was overexpressed, and the high expression of GNGT1 was significantly associated with worse OS in patients with NSCLC. NPSR1 is a G-protein-coupled receptor that induces intracellular signaling upon stimulation by neuropeptide S (NPS) via mobilization of calcium, increased cyclic adenosine monophospate (cAMP) levels, and activation of the mitogen-activated protein kinase (MAPK) pathway [54,55]. Zhang et al. [53] found that the expression level of NPSR1 in NSCLC was overexpressed. In addition, a previous study suggested that NPSR1 is a marker widely expressed in neuroendocrine tumors (NET) with the exception of adrenal pheochromocytomas, the stimulation of NPSR1 with NPS results in activation of pathways that are relevant for cancer development [56].

Considering the enrichment results of the 6 hub genes in the present study, it was demonstrated that LUAD was associated with E2F targets, G2M checkpoint, glycolysis, mitotic spindle, and PI3K-Akt-mTOR signaling. E2F family members play a major role in cell cycle regulation and DNA synthesis in mammalian cells [57]. Previous studies reported that the expression levels of E2Fs were deregulated in several human malignancies, including lung cancer [58,59], breast cancer [60], and gastrointestinal cancer [61]. The G2M checkpoint is initiated to allow repair of DNA damage prior to mitosis. Therefore, G2M checkpoint abnormal is closely related to genomic instability and induce cells undergoing malignant transformation [62,63]. It has been found that the metabolic switch from mitochondrial respiration to glycolysis during hypoxia (where oxidative phosphorylation will be inactive) as well as mitochondrial dysfunction [64,65] are critical for cancer cell growth. One form of genomic instability found in cancer cells, chromosomal instability, is characterized by losses or gains of chromosomes during cell replication [66]. Some studies suggested that chromosomal instability results from a defective mitotic spindle mechanism that allows segregation of improperly aligned chromosomes during mitosis [67]. Marinov et al. [68] found continuous Akt activation and mTOR phosphorylation in 51% of NSCLC samples and 74% of NSCLC cell lines. The release of regulation of PI3K/Akt/mTOR signal transduction pathway can promote the development of lung cancer, while the application of PI3K inhibitors such as LY294002 can promote NSCLC apoptosis [69].

In addition, we also use GEO database to verify the above results. We found 77 DEGs by screening common differentially expressed genes from GSE118370, GSE136043, and GSE140797 datasets. The function of 77 DEGs was mainly enriched in cell adhesion. Furthermore, the enriched KEGG pathways of 77 DEGs included ECM-receptor interaction, dilated cardiomyopathy, focal adhesion, and PI3K-Akt signaling pathway. Subsequently, we screened out 8 genes through the establishment of PPI network and the application of Cytoscape plugin MCODE, which includes the 6 genes screened from TCGA database. So the 6 genes are used as hub genes for further research.

Subsequently, cBioPortal was used to summarize the possible genetic alterations for 6 hub genes in LUAD. We found that the mutation is the most common mutation in 6 hub genes. Mutations in transcription factors have long been known to contribute to tumorigenesis, and previous studies indicated that overexpressed oncogenic transcription factors could alter the core autoregulatory circuitry of the cell [70]. Mutations in a variety of chromatin regulators have been implicated in development of cancer cells, and the normal functions of these regulators provided some clues to the mechanisms involved in altered gene expression. Loss of function mutations in several nucleosome remodeling proteins are associated with multiple types of cancer [71,72]. In addition, ADCY8, ADRB2, CALCA, GCG, GNGT1, and NPSR1 had genetic alterations, including missense mutation, splice mutation, truncating mutation, fusion, amplification, and deep deletion. However, the clinical potential of these genetic alterations needs to be confirmed with larger sample size and the exact mechanism of these genetic alterations also required *in vitro* and *in vivo* verification.

In this paper, we used UALCAN database, RT-PCR, and HPA database to study the expression differences of 6 hub genes between LUAD tissues and normal tissues. The results showed that the expression levels of GCG, GNGT1, NPSR1, and CALCA were higher in LUAD tissues than in normal lung tissues, and the expression levels of ADCY8 and ADRB2 were lower in LUAD tissues than in normal lung tissues. Previous studies have shown that altered expression levels of the 6 hub genes. For example, ADCY8 hypermethylation and altered expression have been observed in endometrial cancer [41,73]. Feigelson et al. [43] showed that high ADRB2 expression promoted the occurrence and development of breast cancer. Lei et al. [47] reported that the expression level of CALCA was positively correlated with cervical lesion pathogenesis. Vangoitsenhoven et al. [51] found that GLP-1, encoded by GCG gene, could lead to pancreatic and medullary thyroid carcinoma. Pulkkinen et al. [56] reported that NPSR1 was a marker widely expressed in neuroendocrine tumor and activated intracellular pathways relevant for cell growth.

During the survival analysis of the present study, KMplot and TCGA database were used to assess the effect of expression levels of the 6 hub genes in patients with LUAD. Notably, high expression of ADCY8 and ADRB2 and low expression of CALCA, GCG, GNGT1, and NPSR1 were correlated with significantly better OS and PFS in LUAD patients. Previous studies have reported the relationship between some of these genes and prognosis of cancers. For example, Wu et al. [74] found that the high expression of ADRB2 was positively relative with the prognosis of hepatocellular carcinoma. Martinelli et al. [75] found that high frequency of CALCA methylation was associated with non-seminomatous tumors and promoter methylation of CALCA predicts poor clinical outcome for testicular germ cell tumors patients. Zhang et al. [53] found that the expression level of GNGT1 in NSCLC was high, and the high expression of GNGT1 was significantly associated with worse OS in patients with NSCLC.

Finally, we analyzed the clinical data of TCGA database to clarify the relationship between the expression level of 6 hub genes and the clinical manifestations of patients with LUAD. The results showed that the expression level of ADCY8 was negatively correlated with T stage, distant metastasis, and TNM stage. The expression level of ADRB2 in female was significantly higher than in male, and the expression level of ADRB2 was negatively correlated with T stage, nodal metastasis, distant metastasis, and TNM stage. The expression level of CALCA in male was significantly lower than in female, and the expression level of CALCA was positively correlated with nodal metastasis, distant metastasis, and TNM stage. The expression level of GCG was negatively correlated with age, and the expression level of GCG was positively correlated with nodal metastasis, distant metastasis, and TNM stage. The expression level of GNGT1 was positively correlated with T stage and nodal metastasis. The expression level of NPSR1 was positively correlated with T stage, nodal metastasis, and TNM stage.

In summary, it can be seen that the expression levels of ADCY8, ADRB2, CALCA, GCG, GNGT1, and NPSR1 are significantly related to the OS and PFS of LUAD, which are also important indicators for the evaluation of the prognosis of LUAD and the evaluation of further treatment. The analysis of the 6 genes indicated that they could effectively distinguish between LUAD tissues and normal tissues, which may increase the accuracy of predicting LUAD.

## Conclusion

In conclusion, ADCY8, ADRB2, CALCA, GCG, GNGT1, and NPSR1 may be potential biomarkers and therapeutic targets for LUAD.

## Data Availability

High-throughput gene expression data of LUAD and normal lung tissues were extracted from the TCGA Data Portal (https://tcga-data.nci.nih.gov/tcga). These RNA-seq data (HTSeq-count) from Illumina HiSeq RNASeq platform consisted of 502 LUAD samples and 49 adjacent non-cancerous lung tissues, and were achieved from the publicly available Genomic Data Commons (GDC) data portal (https://portal.gdc.cancer.gov/). In addition, gene expression profiles of datasets GSE118370, GSE136043, and GSE140797 were obtained from GEO.

## Abbreviations

ADCY8: adenylate cyclase 8
ADRB2: adrenoceptor beta 2
AUC: area under receiver operating characteristics
BH: Benjamini and Hochberg
BP: biological process
CALCA: calcitonin-related polypeptide alpha
CC: cellular component
CI: confidence intervals
DAVID: Database for annotation, visualization and integrated discovery
DEGs: differentially expressed genes
FDR: false discovery rate
GCG: Proglucagon gene
GDC: Genomic data commons
GEO: Gene Expression Omnibus
GNGT1: Transducin γ-subunit gene
GO: Gene ontology
GSEA: Gene set enrichment analysis
IARC: International agency for research on cancer
IHC: immunohistochemistry
KEGG: Kyoto encyclopedia of genes and genomes
LUAD: lung adenocarcinoma
MCODE: molecular complex detection
MF: molecular function
NPSR1: neuropeptide S receptor 1
NSCLC: non-small cell lung cancer
OR: odds ratio
OS: overall survival
PFS: progression-free survival
PPI: protein–protein interaction
qRT-PCR: quantitative real-time PCR
ROC: receiver operating characteristic
STRING: Search tool for the retrieval of interacting genes
TCGA: The Cancer Genome Atlas
